# INTEnsive ambulance-delivered blood pressure Reduction in hyper-ACute stroke Trial (INTERACT4): study protocol for a randomized controlled trial

**DOI:** 10.1186/s13063-021-05860-y

**Published:** 2021-12-06

**Authors:** Lili Song, Chen Chen, Xiaoying Chen, Yijia Guo, Feifeng Liu, Yapeng Lin, Laurent Billot, Qiang Li, Hueiming Liu, Lei Si, Menglu Ouyang, Hisatomi Arima, Philip M. Bath, Gary A. Ford, Thompson Robinson, Else Charlotte Sandset, Jeffrey L. Saver, Nikola Sprigg, H. Bart van der Worp, Chunfang Zhang, Jie Yang, Gang Li, Craig S. Anderson, for the INTERACT4 investigators, C. S. Anderson, G. Li, J. Yang, L. Song, H. Arima, P. Bath, T. Robinson, G. Ford, N. Sprigg, E. Sandset, J. Saver, B. Worp, Z. Liu, J. Dawson, L. Wong, B. Peng, L. Billot, Q. Li, Y. Ning, L. Song, X. Chen, Z. Yang, W. Yu, G. Li, C. Chen, F. Liu, J. Yang, Y. Guo, Y. Lin, R. Hu, H. Cheng, W. Ma, K. Liao, C. Zhang, H. Jin, G. Li, J. Yang, P. Xu, X. Wu, F. Wang, L. Wu, C. Wang, Y. Peng, X. Zhao, X. Xu, X. Liu, X. Song, Z. Li, H. Zhang, D. Yu, Y. Wang, X. Tang, H. Liu, X. Cao, Y. Liu, Y. Duan, H. Liu, C. Li, J. Huang, H. Li, C. Fan, Y. Tang, Y. Ji, G. Li, Y. Huang, G. Chen, J. Wang

**Affiliations:** 1 The George Institute China, Peking University Health Science Center, Room 052A, Unit 1, Tayuan Diplomatic Office Building, No. 14 Liangmahe Nan Lu, Chaoyang District, Beijing, China; 2 The George Institute for Global Health, Faculty of Medicine, UNSW, Level 5, 1 King Street, PO Box M201, 83-117 Missenden Road, Camperdown, NSW 2050 Australia; 3 Department of Neurology, Shanghai East Hospital, School of Medicine, Tongji University, 1800, Yuntai Road, Shanghai, 200120 China; 4 Department of Neurology, Clinical Medical College and The First Affiliated Hospital of Chengdu Medical College, 278, middle section of Baoguang Avenue, Xindu District, Chengdu City, 610500 Sichuan Province China; 5 Department of Preventive Medicine and Public Health, Faculty of Medicine, Fukuoka University, Fukuoka, Japan; 6 Stroke Trials Unit, Mental Health & Clinical Neuroscience, University of Nottingham, Nottingham, UK; 7 Oxford University Hospitals NHS Foundation Trust and University of Oxford, Oxford, UK; 8 College of Life Sciences and NIHR Leicester Biomedical Research Centre, University of Leicester, Leicester, UK; 9 Department of Neurology, Oslo University Hospital, Oslo, Norway; 10 The Norwegian Air Ambulance Foundation, Bodø, Norway; 11 University of California, Los Angeles, USA; 12 University Medical Center, Utrecht, the Netherlands; 13 Shanghai Pudong New Area Medical Emergency Center, Shanghai, China; 14 Department of Neurology, Sichuan Academy of Medical Sciences & Sichuan Provincial People’s Hospital, 32# W. Sec 2, 1st Ring Road, Chengdu, 610072 China; 15 Sydney Medical School, University of Sydney, Sydney, Australia

**Keywords:** Stroke, Pre-hospital, Blood pressure, Ambulance, Management, Clinical trial

## Abstract

**Background:**

Early pre-hospital initiation of blood pressure (BP) lowering could improve outcomes for patients with acute stroke, by reducing hematoma expansion in intracerebral hemorrhage (ICH), and time to reperfusion treatment and risk of intracranial hemorrhage in ischemic stroke (IS). We present the design of the fourth INTEnsive ambulance-delivered blood pressure Reduction in hyper-ACute stroke Trial (INTERACT4).

**Methods:**

A multi-center, ambulance-delivered, prospective, randomized, open-label, blinded endpoint (PROBE) assessed trial of pre-hospital BP lowering in 3116 hypertensive patients with suspected acute stroke at 50+ sites in China. Patients are randomized through a mobile phone digital system to intensive BP lowering to a target systolic BP of < 140 mmHg within 30 min, or guideline-recommended BP management according to local protocols. After the collection of in-hospital clinical and management data and 7-day outcomes, trained blinded assessors conduct telephone or face-to-face assessments of physical function and health-related quality of life in participants at 90 days. The primary outcome is the physical function on the modified Rankin scale at 90 days, analyzed as an ordinal outcome with 7 categories. The sample size was estimated to provide 90% power (*α* = 0.05) to detect a 22% reduction in the odds of a worse functional outcome using ordinal logistic regression.

**Discussion:**

INTERACT4 is a pragmatic clinical trial to provide reliable evidence on the effectiveness and safety of ambulance-delivered hyperacute BP lowering in patients with suspected acute stroke.

**Trial registration:**

ClinicalTrials.gov NCT03790800. Registered on 2 January 2019; Chinese Trial Registry ChiCTR1900020534. Registered on 7 January 2019. All items can be found in this protocol paper.

**Supplementary Information:**

The online version contains supplementary material available at 10.1186/s13063-021-05860-y.

## Administrative information

Note: The numbers in curly brackets in this protocol refer to SPIRIT checklist item numbers. The order of the items has been modified to group similar items (see http://www.equator-network.org/reporting-guidelines/spirit-2727-statement-defining-standard-protocol-items-for-clinical-trials/).

**Table:** 

**Title {1}**	INTEnsive ambulance-delivered blood pressure Reduction in hyper-ACute stroke Trial (INTERACT4): study protocol for a randomized controlled trial
**Trial registration {2a and 2b}**	ClinicalTrials.gov identifier: NCT03790800. Chinese Trial Registry identifier: ChiCTR1900020534.
**Protocol version {3}**	Version 2.0 – 19 July 2019
**Funding {4}**	Program Grant from the National Health and Medical Research Council (NHMRC) of Australia (APP1149987); a seed grant for research in under-served population of low-middle income countries from The George Institute For Global Health; internal grants from Shanghai East Hospital of Tongji University, including Shanghai Key Clinical Discipline, Construction Project of Key Discipline Groups of Shanghai Pudong Health Bureau (No. PWZxq2017-08), Pilot Program of East Hospital Affiliated to Tongji University (2017), Stroke and dementia special fund of Shanghai Science and Technology Development Foundation; International Science and Technology Cooperation Project (2020-GH02-00057-HZ) from Chengdu Science and Technology Bureau; internal grants including Project of Neurology Key Discipline of Sichuan (No.[2018]53), and high-level talent start-up fund (CYFY-GQ10) from Clinical Medical College and the First Affiliated Hospital of Chengdu Medical College, China; and Takeda China.
**Author details {5a}**	^1^The George Institute China, Peking University Health Science Center, Beijing, China ^2^The George Institute for Global Health, Faculty of Medicine, University of New South Wales, Sydney, Australia ^3^Shanghai East Hospital, School of Medicine, Tongji University, Shanghai, China ^4^Clinical Medical College and The First Affiliated Hospital of Chengdu Medical College, Chengdu, China ^5^Faculty of Medicine, Fukuoka University, Fukuoka, Japan ^6^University of Nottingham, Nottingham, UK ^7^Oxford University Hospitals NHS Foundation Trust and University of Oxford, Oxford UK ^8^University of Leicester and NIHR Biomedical Research Centre, Leicester, UK ^9^Oslo University Hospital, Oslo, Norway ^10^ The Norwegian Air Ambulance Foundation, Norway ^11^University of California, Los Angeles, USA ^12^University Medical Center Utrecht, Utrecht, The Netherlands ^13^Shanghai Pudong New Area Medical Emergency Center, Shanghai, China ^14^Sichuan Academy of Medical Sciences & Sichuan Provincial People’s Hospital, Chengdu, China ^15^University of Sydney, Sydney, Australia
**Name and contact information for the trial sponsor {5b}**	The George Institute for Global Health (Australia) Beijing Representative Office, Room 011, Unit 2, Tayuan Diplomatic Office Building, No. 14 Liangmahe Nan Lu, Chaoyang District, Beijing, China. Phone: + 86 10 8280 0577; Fax: +86 10 8280 0177; Email: interact4@georgeinstitute.org.cn Shanghai East Hospital, No.1800 Yuntai Road, Shanghai, China. T: +8613621691786,E: ligang@tongji.edu.cn The First Affiliated Hospital of Chengdu Medical College, 278, middle section of Baoguang Avenue, Xindu District, Chengdu City, Sichuan Province, 610500, China. T: +86 13678130516 E: yangjie1126@163.com
**Role of sponsor and funder {5c}**	The study sponsors fully conduct the design, execution, analysis, interpretation of data, and decision to submit results for this study. The study funders had no role in the design, execution, analysis, interpretation of data, or decision to submit results for this study.

## Introduction

### Background and rationale {6a}

Despite stroke being a major cause of loss in disability-adjusted life-years (DALYs) [1], there are few proven treatments and most are limited by short therapeutic time windows [2, 3]. Intensive blood pressure (BP) lowering is an attractive treatment, as several low-cost antihypertensive agents are widely available, and there is strong epidemiological data supporting the frequent occurrence [4] and prognostic significance of hypertension in both ischemic stroke (IS) or intracerebral hemorrhage (ICH) [5]. However, two of the largest trials of intensive BP lowering in acute ICH had inconsistent effects on functional recovery, despite the treatment being shown to be safe and able to attenuate hematoma growth [6–8]. Similarly, the Enhanced Control of Hypertension and Thrombolysis Stroke Study (ENCHANTED) showed that intensive BP lowering specifically in patients thrombolyzed for acute IS failed to improve functional recovery despite reducing the risk of intracranial hemorrhage [9]. There is also uncertainty over the optimal level of BP control in patients with IS due to large vessel occlusion treated with mechanical thrombectomy [10].

Despite these caveats, use of BP lowering in the pre-hospital setting has attracted attention in potentially being able to reduce hematoma expansion in ICH, and shorten the time to initiate reperfusion therapy and reduce subsequent risk of intracranial hemorrhage in IS. However, concerns have been raised by results of the second Rapid Intervention with Glyceryl trinitrate in Hypertensive stroke Trial (RIGHT-2). Not only was there no overall improvement in functional outcome from the use of a transdermal glyceryl trinitrate (GTN) patch in patients with presumed stroke, but there were worse outcomes in those with a final diagnosis of ICH [11]. This work has highlighted the potential for enhanced bleeding in relation to vasodilation and antiplatelet effects of GTN and possibly other BP lowering agents [12], as well as fuelling debate over the promotion of cerebral ischemia within the vulnerable penumbra of IS from such an approach [13].

Thus, we initiated the fourth in a series of early intensive BP lowering trials, the INTEnsive ambulance-delivered blood pressure Reduction in hyper-ACute stroke Trial (INTERACT4), as a pragmatic, multi-center, ambulance-delivered, prospective, randomized, open-label, blinded endpoint assessment (PROBE) study to determine the effectiveness and safety of hyperacute BP lowering for suspected acute stroke in China. Herein, we report the final version of the trial protocol, compliant with the Standard Protocol Items: Recommendations for Interventional Trials (SPIRIT) reporting guideline, with sub-title labeled by SPIRIT item number.

## Objectives {7}

### Hypothesis

Hyperacute intensive BP lowering initiated in the ambulance can improve functional outcome in patients with suspected acute stroke.

### Research questions

Compared to guideline-recommended BP management, does hyperacute intensive BP lowering initiated in the ambulance:

1. Improve functional outcome in patients with acute stroke?
2. Prove to be safe in all patients with suspected acute stroke?
3. Reduce the likelihood of death, death or dependency, and duration of hospitalization and improve physical function, health-related quality of life (HRQoL), and living circumstances?
4. Reduce hematoma expansion specifically in patients with a final diagnosis of ICH?
5. Increase access to reperfusion therapies (thrombolysis and/or thrombectomy) in shorter time from symptom onset and reduce the risk of intracranial hemorrhage and size of cerebral infarction, specifically in patients with IS?
6. Show no heterogeneity in the treatment effect across certain types of patients?
7. Provide cost benefits?

## Trial design {8}

**Fig. 1:**
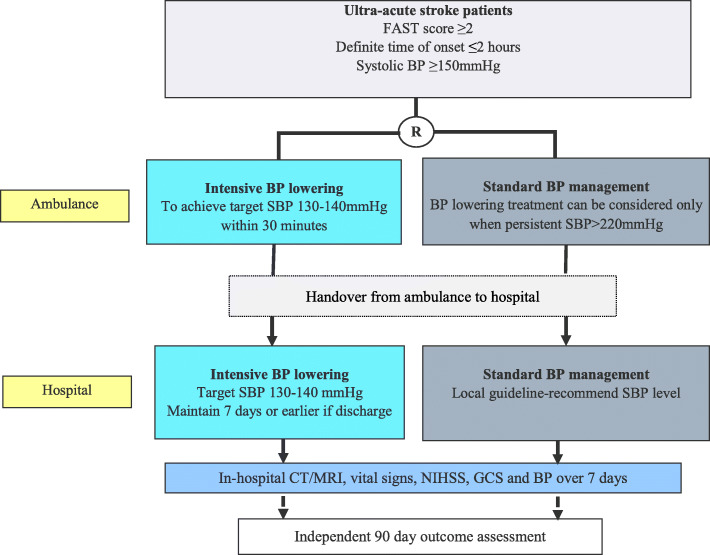
Study flow chart. Footnote: BP indicates blood pressure, CT computerized tomography, FAST Face/Arm/Speech/Time scale, GCS Glasgow coma scale, MRI magnetic resonance imaging, NIHSS National Institute of Health Stroke Scale, SBP systolic blood pressure

The INTERACT4 study is a multi-center, ambulance-delivered, prospective, randomized controlled, open-label, blinded outcome assessed (PROBE) trial conducted through a network of regional hospital clusters, where a total of 3116 patients with suspected acute stroke are planned to be recruited across 50+ hospitals in China. Potentially eligible patients will be recruited by either of two mechanisms: (i) waiver of consent to the intervention in the ambulance, and written consent for follow-up obtained in hospital; or (ii) consent to the intervention via a brief written consent form in the ambulance, and written consent for follow-up in hospital (if a waiver of consent is not approved by the local ethics committee). Randomized allocation of the intervention will be done in a 1:1 ratio, using a central, automated, mobile-phone accessed mini-program electronic software, with stratification based on region, age (≥ 65 vs < 65 years) and Face/Arm/Speech/Time (FAST) deficit severity score (score 3-4 vs 2). Ambulance staff are trained in the study protocol, covering approaches to assessment, randomization, treatment, and collection of key baseline data. All information in ambulance, including basic demographics, randomized treatment allocation, and BP measurement and treatment details, are collected through the mobile electronic database system which is connected to investigator clinicians at local/regional hospitals. Other relevant documents, such as the consent forms and BP charts, are handed over to hospital investigators as part of the transfer of care process. Endpoint assessments are blinded to treatment allocation. A schema of the study design is provided (Fig. 1).

## Methods: participants, interventions, and outcomes

### Study setting {9}

The trial is being conducted at approximately 3–4 medical emergency centers (MECs) that provide ambulance services linked to hospitals in China covering an eastern area of high-level of resources (i.e., Shanghai, Jiangsu and Shandong provinces) and western and northern areas with lower levels of resources (i.e., Sichuan, Yunnan, Shanxi, and Inner Mongolia). A full list of collaborators can be found in Additional file 1. List of INTERACT4 collaborators. The study involves two different types of ambulance administration systems. The first is an independent organization whereby staff (medical and driver) are managed and dispatched from a central MEC to transfer patients to hospitals within a region. This is the situation for the district of Pudong (population 5.5 million over 1400 km^2^) in Shanghai, where the government MEC takes responsibility for the administration of all ambulance and emergency staff in the population which is served by 8 hospitals. The other model of MEC is more common in China, whereby ambulances are owned, and staff (medical and driver) employed and dispatched from individual hospitals within a region. This is the situation for the Chengdu area, where approximately 30 hospitals, each with their own ambulance service, are participating in the study.

### Eligibility criteria {10}

To be eligible for inclusion, patients need to satisfy all the following criteria:

- Age ≥ 18 years
- Acute syndrome due to presumed acute stroke, defined by a FAST score of 2 or 3 with an arm motor deficit, and a time ≤ 2 h from last seen well
- Systolic BP ≥150 mmHg(recorded twice)
- Able to provide brief informed consent (if waiver of consent is not approved by the relevant ethics committee)

Patients will not be eligible if there is one or more of the following:

- Coma—no response to tactile stimuli or verbal stimuli (e.g., Glasgow coma score < 5)
- Known severe co-morbid disease (e.g., cancer, chronic airflow disease, severe dementia, severe heart failure, pre-stroke disability [i.e., needing help with daily activities])
- History of epilepsy or seizure at the onset of symptoms
- History of recent head injury (< 7 days)
- Hypoglycemia (glucose < 2.8 mmol/L)

In each case, the decision about a patient’s eligibility will be based on the interpretation of these criteria by participating ambulance staff.

### What is the consent process? {26a}

Each participating site must obtain written approval(s) from their hospital research ethics committee (EC) (e.g., institutional review board [IRB]), and any other relevant regional or national body, before patient recruitment commences. A two option, mixed consent process is the proposed method for use in the study, according to local/national rules and regulations.

Option 1: (a) Waiver of consent for administration of study intervention in the ambulance and (b) consent for use of medical data and follow-up of patients obtained in hospital.

Reasons why waiver of consent is requested for this study are outlined below.

- Ambulance staff have limited time to obtain consent during their assessment, retrieval, and rapid transport of a patient to the hospital. Taking additional time to obtain informed consent could delay a patient’s arrival at hospital, and potentially compromise their management and outcome.
- Stroke is a critical illness, with a proven limited time window for reperfusion treatments to benefit a patient with IS and a hypothesized limited time window for BP moderating treatments to benefit a patient with ICH. The trial is assessing the benefits of rapid BP lowering treatment initiated as soon as possible after the onset of symptoms. Taking additional time to obtain informed consent can delay the initiation of treatment and reduce potential benefits to the patient.
- Many patients with acute stroke do not have cognitive capacity to provide informed consent, due to impaired consciousness, aphasia, or confusion. Limiting participation only to patients with mild symptoms reduces the ability to assess the treatment in a broad range of patients, and in particularly those with more severe illness who have the greatest potential to benefit from reducing initial and secondary brain injury after ICH and/or IS.
- Waiver of consent is often applied in the prehospital setting for other similar critical illnesses, such as cardiac arrest, head injury, and status epilepticus.
- The requirement for consent may compromise the relationship between the ambulance staff and patient/family, create suspicion/concern and/or refusal to receive standard care, and potential to worsen patient outcomes.
- The study intervention is low risk, as the recommended BP lowering agent—urapidil—is readily available in clinical practice and is being used within its licensed indication to treat acute hypertension. Urapidil has a well-accepted low adverse event profile, with the most common side-effect of hypotension being readily corrected through its discontinuation and the use of intravenous (IV) fluid replacement, for which ambulance staff are well trained in its management. The administration of urapidil is by trained and accredited ambulance-assigned doctors, and all adverse events will be systematically reported to, and monitored by, project staff; and reported and reviewed by an independent Data Safety Monitoring Board (DSMB). The BP monitoring protocol has been well tested in patients with acute stroke presenting to hospital, including those with acute ICH and IS, where the small risk of harms being well defined.

Option 2: (a) Brief (limited) informed consent in ambulance (if waiver of consent is not approved by the EC) followed by (b) consent for use of medical data and follow-up of patients obtained in hospital.

A brief consent will be obtained at the stroke scene or in the ambulance, where four simple items will be contained in the consent form and explained by the ambulance doctor to the patient: (i) they are suspected to have acute stroke, (ii) their BP may benefit from lowering as early as possible, (iii) that IV bolus of a drug to lower the BP or usual treatment without ambulance BP lowering will be applied, and (iv) do they agree to take part in a research study. If the patient is not fully competent to give informed consent (e.g., aphasia or reduced level of consciousness), the patient’s next-of-kin or surrogate will be approached to sign this consent form on his/her behalf.

If brief (limited) informed consent in the ambulance is approved by local EC, for noncompetent patients for whom there is no next-of-kin present to provide informed consent, further approval will be sought to allow ambulance staff to proceed in randomizing a patient using the physician-judgment consent method: an ambulance officer will sign on behalf of the patient, if there is no next-of-kin present and the patient is not competent to provide informed consent.

All competent randomized patients will be given a patient information sheet and informed consent form (PISCF) as soon as possible following their admission to hospital, ideally within the first 72 h. The PISCF will outline that the hospital (site) is “participating in research that is assessing early BP control delivered in ambulance on progression of the stroke and recovery” and that there is a need for data collection (in-hospital) and follow-up assessment at 3 months. The patient will have the opportunity to discuss and seek explanation from an approved clinician investigator familiar with the study protocol and use the locally approved consent process for collection of data (in-hospital and/or follow-up assessments).

In many patients with acute stroke, symptoms of the stroke can include diminished level of alertness, impaired language, confusion, and other cognitive deficits that render the patient unable to comprehend much of the information that is given to them. If a patient is not fully competent, the patient’s “surrogate” will be approached and will be provided with the PISCF to read and act on the patient’s behalf. A “person responsible” is the legally appointed guardian, their spouse or de-facto spouse or same sex partner; or if there is none, their unpaid carer; or if there is none, their relative or friend who has a close relationship with the person.

In situations where the patient is unable to provide consent and consent has been obtained from their surrogate, the patient will be made aware of this as soon as he/she is well enough, ideally before 7 days or their discharge from hospital, so that he/she will have an opportunity to discuss and seek explanation from a clinician investigator associated with the study. Patients will be given the opportunity to re-consent for the collection of their data (in-hospital and/or follow-up assessments) during their stay in hospital, or as soon as they are able to decide, according to the locally approved consent process.

If the patient is dying or remains unable to record their personal consent during follow-up, the consent given by the representative will stand, and study data will be retained (or removed, if appropriate). The reason for not being able to obtain the patient’s consent will be documented, dated, and signed, and included in the patient’s medical record for filing.

Withdrawal of consent: The PISCF provided to the patient and/or the next of kin or surrogate will clearly state that the patient can be withdrawn from the study at any time without prejudice and explanation. Such withdrawal should be documented in the patient’s file. If withdrawal of consent relates to the BP management alone, data collection can continue provided there is documentation of this fact in the patient’s files.

### Additional consent provisions for collection and use of participant data and biological specimens {26b}

Not applicable.

### Interventions

#### Explanation of the choice of comparators {6b}

The active comparator (intervention) is a treatment strategy of intensive BP lowering initiated in the ambulance in those participants with suspected stroke and hypertension to achieve a systolic BP target of < 140 mmHg within 30 min, and to maintain this BP level for the next 7 days, or at the time of hospital discharge should this occur earlier. The control comparator (control) is the treatment strategy according to local protocols based upon guideline-recommended BP management, both in the ambulance and the hospital. For those participants allocated to control group, BP lowering treatment can be considered for use in ambulance only in those patients with persistently very high systolic BP (e.g., ≥ 220 mmHg) or diastolic BP (e.g., ≥ 110 mmHg). After admission to hospital, patients in the control group should be treated based on a local guideline of each participating hospital, the features of which will be delineated.

#### Intervention description {11a}

The intervention, intensive BP management initiated in the ambulance, is to commence as early as possible after assessment of a patient, and to achieve a systolic BP level < 140 mmHg within 30 min, and to maintain this BP level for the next 7 days, or the time of hospital discharge should this occur earlier.

A recommended treatment regime is giving an IV bolus of 25 mg urapidil administered over 1 min, and another 25 mg urapidil bolus if the BP level persists > 150 mmHg after 5 min. Urapidil is a sympatholytic antihypertensive drug, which acts both as an α_1_-adrenoceptor antagonist and a 5-HT1A receptor agonist, and has a rapid onset (5 min, maximum at 15–30 min) and no effect on intracranial pressure (ICP). BP monitoring in the ambulance will occur every 5 min on electronic charts, delivered to the hospital staff. Allocation to early intensive BP lowering will be notified to hospital investigators as early as possible by the ambulance staff. Patients should keep a horizontal position while being transported to the hospital or other location. IV bolus (or maintenance infusion) treatment with urapidil will continue in the emergency department (ED), where it is anticipated that patients will stay (except for acute CT or MR imaging) until they are clinically stable, and that the target BP is achieved and maintained. BP lowering treatment chosen by the treating clinician will be continued in an acute stroke unit or other monitored facility, to maintain a systolic BP level < 140 mmHg for the next 7 days. Oral BP lowering agents (dependent upon local availability) can be used after a stable target BP level is reached, but it is expected that IV therapy will continue to be required during the initiation of oral antihypertensive therapy, to maintain the systolic BP levels of < 140 mmHg. A systolic BP < 130 mmHg is considered the threshold for cessation of therapy. Each site receives a standardized, stepped titratable, intravenous BP lowering protocol, based upon available medications, that is established in advance.

#### Criteria for discontinuing or modifying allocated interventions {11b}

The investigator must not deviate from the protocol, except where the patient/surrogate chooses to withdraw the consent of participation in this study. However, allocated management in either group should be discontinued or modified if any of the following occur: (i) a serious adverse event (SAE), which in the opinion of the investigator, is related to the trial protocol and (ii) the investigator feels that it is in the participant’s best interest.

Follow-up data will be collected for all participants, except those who specifically withdraw.

#### Strategies to improve adherence to the intervention {11c}

A process evaluation, designed to gain insights into the barriers and facilitators to change pre-hospital management and implementation of the intervention, will be undertaken through formative stakeholder interviews alongside with the trial. Preliminary findings have highlighted various barriers to BP lowering, whereby many ambulance doctors (i) are initially cautious with the treatment due to concerns over adverse effects, (ii) are not familiar with using IV urapidil, and (iii) have problems with continuity of the treatment during the transfer of care over to ED staff on presentation to hospital. To address these barriers, regular SAE reports related to intervention and DSMB recommendations are provided to both ambulance and hospital investigators, to inform them of the safety of intensive BP lowering in the hyperacute phase of stroke. Training on the administration and effects of urapidil and other rapid BP lowering agents are given to ambulance doctors. Regional coordination center (RCC) project staff will liaise with site investigators to establish the admission and treatment procedures in the ED at each site. Finally, ambulance and site investigators will be provided regular intervention quality reports during the study, as well as remote communication and on-site monitoring to improve the adherence to the intervention.

#### Relevant concomitant care permitted or prohibited during the trial {11d}

All medical or surgical treatments, besides the allocated BP management, are permitted during the study; they are to be recorded on the case report form (CRF).

#### Provisions for post-trial care {30}

Not applicable. This study is evaluating the effectiveness of intensive BP lowering commenced in the ambulance in patients with suspected hyperacute stroke. The result may modify current medical practice, in particular over pre-hospital assessment and care. However, as this intervention is already available in routine practice, it can be sustained beyond completion of the study.

### Outcomes {12}

The primary outcome is functional recovery on the modified Rankin scale (mRS) measured by structured interview at 3 months, with analysis as an ordinal outcome (shift across all seven scoring categories of physical function that range from 0 to 5, with death as 6) [14].

Secondary outcomes include:

• For ICH patients—hematoma volume at presentation and 24 h, with analysis as dichotomized outcomes of relative (> 33%) and absolute (> 6 mL) growth over 24 h

• For IS patients—time to, and rate of, reperfusion treatment (thrombolysis and/or thrombectomy); infarct size on MRI within 2 days after stroke onset; and frequency of symptomatic intracranial hemorrhage, measured centrally with standard definitions

• For all stroke patients—functional outcomes (death or dependency [mRS scores 3-6]) at 3 months; separately on death or dependency at 3 months; death or neurologic deficit progression measured by National Institutes of Health Stroke Scale (NIHSS) scores [15] at 24 h and 7 days, with analysis as a continuous outcome using linear regression with adjustment for baseline score; and length of hospital stay, place of residence, and health-related quality of life (HRQoL) according to the EuroQoL Group 5-Dimension self-report questionnaire (EQ-5D) [16], all at 3 months

Safety outcomes in all participants including those with a transient ischemic attack and stroke mimic will be recorded for the duration of follow-up. They will be assessed as all-cause and cause-specific SAEs and categorized according to standard organ system-specific criteria.

### Participant timeline {13}

The schedule of randomization for sites as well as enrolment, treatment allocation, and assessments for participants is outlined in Table 1.

**Table 1:** Collection of data in INTERACT4

Evaluation	Screen + enrolment log	Baseline	Day 1	7 days/discharge/transfer/death^**a**^	BP monitoring chart	3-month follow-up
Forms	A	B	C	D	E	F
Eligibility	X					
Consent/re-consent	X			X		
Vital signs	X	X	X	X		
BP	X^b^	X	X	X	X^c^	
GCS		X	X	X		
NIHSS		X	X	X		
Medical history		X				
CT scan		X	X	X		
MRI scan for AIS			X			
mRS		X		X		X
EQ-5D						X
Routine blood tests		X		X		
Standard stroke care		X		X		
Final diagnosis				X		
Medications in use			X	X		X
SAEs			X	X		X
Healthcare cost	X			X		X

*Abbreviations: AIS* acute ischemic stroke, *BP* blood pressure, *CT* computerized tomography, *EQ-5D* EuroQol 5 dimensions instrument for assessment of health-related quality of life, *GCS* Glasgow coma scale, *MRI* magnetic resonance imaging, *mRS* modified Rankin scale, *NIHSS* National Institute of Health stroke scale, *SAEs* serious adverse events

^a^Assessed at earliest time point among day 7, discharge, transfer, or death

^b^Q5 min for 30 min after randomization; Q15 for 1 h

^c^Q15 min for 1 h after admission to hospital; hourly from 1 to 6 h after admission to hospital; 6 hourly from 6 to 24 h after admission to hospital. At any point where intravenous bolus drugs are administered, BP and HR are recorded 5 and 15 min later, respectively

### Sample size {14}

The sample size for this study is based on 90% power (*p* = 0.05) to detect a 22% reduction in the odds (common odds ratio [OR] of 0.78) of a worse outcome using an ordinal logistic regression. Assuming a distribution of mRS in the control group that is similar to that observed in the placebo group of the Field Administration of Stroke Therapy–Magnesium (FAST-MAG) trial [17], the largest pre-hospital stroke management trial that included suspected stroke patients within 2 h of symptoms onset (mRS distribution of 18.4%, 16.2%, 18.3%, 13.3%, 10.6%, 10.2%, and 13.0%, for scores of 0 to 6, respectively), it would correspond to a 6.1% absolute improvement in the proportion of patients experiencing a poor outcome (mRS scores 3-6), that is from 47.1% down to 41.0%. This would translate into a 13% relative risk reduction (relative risk of 0.87). Assuming 30% of participating patients will have a stroke mimic and 5% will have missing outcomes, the total sample size is 3116 participants to be recruited over 3 years.

### Recruitment {15}

The study co-investigators, as regional leaders, are responsible for attracting eligible MECs and hospitals from their various networks. High-quality hospitals that have participated in previous studies of The George Institute for Global Health will also been invited to participate. As recruitment occurs in the ambulance, training and screening oversight of the ambulance medical staff is critical to achieving the recruitment target. Regular and specific training on recognition of suspected stroke patients, mastering eligibility criteria, and using the randomization system on smartphone will be provided by RCC staff. All suspected stroke patients are required to be entered into this mini-program, as well as details of the reasons why screen-fail patients are excluded from the trial. A screening report will be given to both the Central Coordinating Center (CCC) and RCC.

## Assignment of intervention: allocation

### Sequence generation {16a}

An internet-based electronic randomization system will be accessed to allocate eligible participants to randomized BP management group in a 1:1 ratio. The randomization sequence will use an algorithm to ensure balance in key prognostic factors, according to the stratification variables of region, age (≥65 vs < 65 years) and FAST score (3-4 vs 2).

### Concealment mechanism {16b}

Concealment of treatment group assignment until the patient has been randomized will be accomplished by making treatment allocation known only after study personnel have enrolled the patient and entered patient characteristics into the internet-based randomization application.

### Implementation {16c}

After patient eligibility is confirmed, the investigator in the ambulance will access a secure 24/7 internet-based electronic randomization system based upon a Wechat app on a mobile phone developed by Bioknow company. Before randomization, the system requires several stratification factors to be entered including region, age, and FAST score. The investigator will then deliver the allocated treatment to the patient according to the randomization result showed on the mobile phone. All suspected acute stroke patients screened for the study that are not included, as well as recruited patients, must be recorded on the screening/enrolment log. This information is to be uploaded to the CCC database.

## Assignment of interventions: blinding

### Who will be blinded {17a}

The treatment is open label to patients and investigators who need to provide BP management in the ambulance and at sites. All other investigators, statisticians and end-point assessors, who are trained to collect outcome measures by face-to-face visit or telephone at 3 months, are blind to the treatment allocation.

### Procedures for unblinding if needed {17b}

Not applicable. This study is an open-label intervention.

## Data collection and management

### Plans for assessment and collection of outcomes {18a}

Ambulance investigators are required to collect eligibility data on patients at the stroke scene or in the ambulance. Measurement, monitoring, and treatment related to BP should be recorded on a specific chart provided by study and then handed over to hospital investigators when bridging occurs at hospital sites.

Hospitals are required to collect data on patients at admission (baseline), 24 h and 7 days BP monitoring chart, separation (day 1; day 7 or at discharge if earlier, transfer from the hospital or death), and all SAEs including death until the 3 months of follow-up. The follow-up assessments are to be undertaken by an investigator who was not involved in the clinical management of the patient, and blind to the study treatment allocation.

Study management will be facilitated by an established internet-based system. Table 1 illustrates the schedule and nature of the data collection required during the study period.

### Plans to promote participant retention and complete follow-up {18b}

The investigators collect a range of contact information, including those of the patient and several relatives and/or friends at hospitalization. The investigators emphasize to participating patients (or their responsible person[s]) during the consent process and at discharge, that they are to receive a telephone call at 3 months to invite them coming back to outpatient to check on their health status face-to-face. If the patients are unable to go to clinic, the assessors will conduct the assessment via telephone directly.

### Data management {19}

Ambulance investigators record the measurement, monitoring, and treatment related to BP on a specific chart provided by study and handed over to hospital investigators when bridging to sites. Hospital investigators are responsible to enter the records into the electronic database. Sites receive paper versions of the CRFs and a procedure manual to serve as a reference guide in using the database; each data element is defined to ensure investigators are accurate and consistent in data entry. All data entry will be completed via a secure web-based electronic data collection system. This will allow for real-time data query generation for values entered outside of pre-set valid ranges, and for consistency checking. This system will speed up data reporting and assist overall trial management for all participating centers. Data entry will be performed at the participating sites. Only authorized staff will have access. All entered data forms will be electronically signed (by use of the unique password) by authorized study staff. All changes made following the initial entry will have an electronically dated audit trail. Centralized coding of outcomes will be performed by a trained medical coder using Medical Dictionary for Regulatory Activities (MedDRA) criteria: he/she will review reporting of outcomes and confirm the accuracy of coding.

### Confidentiality {27}

Every precaution is taken to respect the privacy of participants in the conduct of the study. Only de-identified data will be used for statistical analysis and the publication of results to maintain confidentiality. During monitoring of data quality and adherence to the study protocol, research staff will refer to source documents (medical records) at participating hospitals. This information is included in the PISCF. All individual and site information will be de-identified in reports and results to further protect the confidentiality of participants.

### Plans for collection, laboratory evaluation, and storage of biological specimens for genetic or molecular analysis in this trial/future use {33}

Not applicable. Biological specimens are not collected as part of this study.

## Statistics methods

### Statistical methods for primary and secondary outcomes {20a}

The intention to treat (ITT) principle will be applied in the main analysis, for all participants with presumed acute stroke as if they had received the intervention to which they were supposed to receive, irrespective of whether or not the treatment was actually received, and regardless of subsequent withdrawal from treatment or deviation from the protocol. The primary endpoint of functional recovery at 90 days will be assessed using the mRS score and analyzed using adjusted ordinal logistic regression. The intervention effect will be estimated as the common odds ratio (OR) and its 95% confidence interval (CI). The model will be adjusted by the stratification variables (region, age, and FAST score) as well as pre-morbid mRS score. In case of violation of the proportional odds assumption, the common OR and 95% CI will still be reported; however, a sensitivity analysis will be undertaken using linear regression and treating the mRS score as a continuous variable, thus estimating the effect of the intervention as an adjusted mean difference in mRS score. As a reference, an unadjusted sensitivity analysis without any baseline covariate being included will also be undertaken. The study will use a hierarchical approach for analysis of the study populations, starting with a primary analysis of all participants with a confirmed stroke (ICH or IS) and progressing to a secondary population of all randomized patients (i.e. including ICH, IS, transient ischemic attack, and mimics). A per-protocol (PP) analysis will also be undertaken only for those patients who strictly adhered to all aspects of the protocol. Multiplicity will be controlled by sequential gatekeeping. The primary population will be analyzed first with p threshold of 0.05. If the primary population shows *p* ≤ 0.05 on the primary outcome analysis, then the secondary population will next be analyzed also in a hypothesis-confirming manner with *p* threshold of 0.05. If the primary population shows *p* > 0.05, then the secondary population will next be analyzed in an exploratory manner (with nominal *p* threshold of 0.05). No further adjustment for multiplicity is planned since all secondary analyzes are hypothesis-generating and designed to support the primary analysis.

Binary endpoints such as death and dependency and early neurological deterioration will be analyzed using adjusted binary logistic regression. Continuous outcomes, such as NIHSS score or changes in hematoma volume, will be analyzed using linear regression that includes the baseline outcome value (e.g. NIHSS score or hematoma volume) as a covariate.

A detailed analysis plan including mock tables will be finalized before the database is locked.

### Interim analyses {21b}

Two “formal interim analysis” meetings will be held by DSMB by teleconference (or face-to-face, if possible) to review data relating to treatment efficacy, patient safety, and quality of trial conduct. A recommendation to discontinue prematurely will be based upon there being clear evidence that the treatment provides protection or causes harm for an important clinical outcome. The DSMB will work on the principle that a difference of at least 3 standard errors in an interim analysis of a major outcome event (e.g., death from all causes or independent survival at 3 months) between patients allocated to the intensive or the control group, to justify halting, or modifying the study, before the planned completion of recruitment. Given the minimal impact of this approach on the type-I error rate, no adjustment is made to the final significance level [18].

### Methods for additional analyses {20b}

#### Health economic evaluation

Health economic evaluation provides value judgment for health policy makers when they consider scaling up the health intervention. In INTERACT4, the intervention of BP lowering will be initiated in the ambulance and the BP target will be maintained during hospitalization for 7 days. The potential for clinical benefit may be offset by heavy workload, shortage and frequent turnover of staff, insufficient professional training, limited medical devices, and variable transportation imposed within the local healthcare system. As half of the proposed participating sites are secondary hospitals with limited resources, including clinical devices and professional staff, it is important to know whether the study intervention is cost effective compared to the conventional care. A within-trial economic evaluation will be conducted to compare the incremental costs, including costs of intervention and difference in health service cost versus the incremental effectiveness, which will be expressed using quality-adjusted life years. Intervention and healthcare costs will be collected from the trial. The incremental cost effectiveness ratio will be calculated and then compared to the willingness-to-pay threshold in China, to determine the cost-effectiveness of the intervention. The economic evaluation will be conducted from the healthcare payer’s perspective.

#### Process evaluation

The intervention is complex and is required to be implemented in the context of managing patients with suspected acute stroke. Implementation of the intervention involves collaboration across different organizational settings (ambulance, ED, neurology/neurosurgical department and intensive care unit [ICU]). It is important to evaluate the quality of implementation and the perspectives of clinicians in the trial in such complex health system context. The fidelity, reach, dose, adoption, feasibility, and appropriateness of the intervention, as well as contextual conditions (current policies, settings resources etc.) that affect the delivery of the intervention, will be evaluated in the trial. The Medical Research Council (MRC) process evaluation framework and normalization process theory (NPT) will be used to generate questions and indicators for the evaluation regarding conceptions to inform implementation strategies and explore how the trial interventions are integrated into routine health care practice [19].

Both qualitative and quantitative data will be used to address these objectives of process evaluation. Focused group discussions will be conducted with ambulance staff to collect information on implementation to understand the fidelity of intervention in the ambulance. To evaluate the intervention implementation in the hospital, semi-structured interviews and non-participants’ observations will be conducted with physicians and nurses from participating sites. Additional data sources such as observational records (such as routine monitoring data, field notes and CRFs) will be obtained to explore the dose and reach of the intervention. The evaluation will be conducted across different time points (both in the early and mid-phases of the study) to determine any issues that the research team can help to address. The sites involved in the process evaluation will be selected by purposive sampling according to pre-specified criteria (e.g., performance, recruitment quality and workload). All qualitative data will be collected by trained interviewers and observers to ensure internal validity and the data will be recorded and transcribed for further analysis under participant’s permission.

### Methods in analysis to handle protocol non-adherence and any statistical methods to handle missing data {20c}

The ITT approach is to be performed as the primary analysis of intervention effect. A sensitivity analysis or per-protocol approach will be conducted to detect the consistency of primary outcome. The population involved in the final analysis will be fully described, and the differences of baseline characteristics between analyzed and enrolled populations will be compared. Any missing primary outcomes at 90 days will be handled using a multi-imputation method incorporating each patient’s baseline variables, and all available neurologic deficits (NIHSS) and global disability (mRS) values.

### Plans to give access to the full protocol, participant-level data, and statistical code {31c}

The data collected is owned by the TSC. Datasets generated and/or analyzed will be available to all study investigators, and to investigators at other institutions around the world, according to a strict data sharing agreement. Data sharing will be available from 12 months after publication of the main results. Investigators are to make a formal request for data sharing through the Global Research Committee of The George Institute for Global Health, and according to a data sharing policy (https://georgeinstitute.sharepoint.com/TGIPolicy/Data%20Sharing%20Policy.pdf). Access will be controlled by the Principal Investigators (PIs) with the approval of the trial steering committee (TSC).

### Publications and reports

Publication of the main reports from the study will be in the name of INTERACT4 Collaborative Investigators. Full editorial control will reside with a Writing Committee approved by the SC. Writing Committees will be formed from members of the various committees, statisticians, research fellows and investigators. Authors of publications must meet International Committee of Medical Journal Editors (ICMJE) guidelines for authorship.

We have designed the study so that the data can be shared to external groups for secondary analysis, such as individual patient data meta-analysis, according to formal data sharing agreements approved by the Research Office of The George Institute as well as regulations and laws of the People’s Republic of China. A full list of secondary analyses will be outlined a priori in the Statistical Analysis Plan (SAP) prior to unblinding of the data.

## Oversight and monitoring

### Composition of the TSC and CCC {5d}

#### TSC

The TSC comprises the PIs, regional leaders, and expert academic researchers in the fields of stroke, neurocritical care, neurology, cardiovascular epidemiology, and clinical trials and is governed by a Charter (Additional file 3). The TSC is responsible for overseeing the execution of the study design, protocol, data collection, and analysis plans, as well as publications.

#### CCC

The CCC is based at The George Institute China, supported by project staff, and is responsible for the day-to-day management of the study, data and project management, committee coordination, assistance with ethics committee and regulatory applications, protocol and procedures for training of participating sites, overseeing of initiation visits and activation of participating centers, monitoring of data quality and adherence to protocol, applicable guidelines and regulations, and preparation of study data for analysis and publication.

### Composition of the data monitoring committee, its role, and reporting structure {21a}

#### DSMB

The DSMB is independent of the sponsor, and responsible for reviewing the safety, ethics, and outcomes of the study. During the period of patient recruitment, the DSMB monitors the primary, secondary, and safety outcomes for early dramatic benefits or potential harmful effects and provides reports to the TSC on recommendations to continue or temporarily halt recruitment to the study. The DSMB is governed by a Charter outlining responsibilities, procedures, and confidentiality and reviews the accumulating unblinded data at regular intervals (Additional file 2).

### SAE reporting and harms {22}

An SAE is defined according to standard convention as any untoward medical occurrence that results in any of the following: (i) results in death, (ii) is life threatening in the opinion of the investigator (at the time of the event), (iii) requires admission to hospital or prolongation of an existing hospital stay, (iv) results in persistent or significant disability or incapacity, (v) results in congenital anomaly or birth defect, or (vi) is an important medical event in the opinion of the investigator that is not immediately life-threatening and does not result in death or hospitalization but which may jeopardize the patient or may require intervention to prevent one of the other listed outcomes.

All SAEs are systematically collected by investigators according to questions outlined in the case report forms (CRF) at each follow-up, and according to the International Conference on Harmonization of Good Clinical Practice (ICH-GCP) guidelines for reporting of SAEs. An SAE form must be used to record the details of the event, and this will include a full description of the event, classification of the event using the above definitions, the PI’s opinion on the causal relationship to the randomized management group, and the timing of the event. The PI will be required to submit at least one follow-up report to provide further information for the outcome of the SAE to be recorded. The SAE should be documented in the medical records or patient file and signed and dated by the investigator, for audit and monitoring. All SAEs should be reported to the CCC within 24 h or as soon as the event is recognized. All SAEs are reviewed by a medical monitor assigned to the trial and coded using Medical Dictionary for Regulatory Activities (MedDRA) criteria. Safety outcomes are reported to the presiding EC in line with their requirements every 6 months, as well as for review by the DMSB at each meeting. All SAEs will be published in the main report.

## Frequency and plans for auditing trial conduct {23}

There are no plans for auditing trial conduct.

## Plans for communicating important protocol amendments to relevant parties (e.g., trial participants, ethics committees) {25}

All protocol amendments are approved by the TSC and communicated to co-investigators and commercial partners. The amended protocol can be implemented only after review and approval by ethics committees.

## Dissemination plans {31a}

In addition to relevant reports developed in formats suitable for various stakeholders, the findings will be published in high impact journals, presented at national and international conferences on stroke, cardiovascular disease, and hypertension. A series of seminars will be held at the end of the study across China, targeting academics, researchers, clinicians, and local health officers. Discussion and debate will assist in integrating the results, whatever the findings, into clinical practice and to influence the decisions of guideline and policy makers.

## Discussion

INTERACT4 is a multi-center, ambulance-initiated, pre- and in-hospital-combined pragmatic clinical trial being conducted in China that aims to address ongoing uncertainties over the effectiveness and safety of early intensive BP lowering in patients with suspected stroke. The participation of a broad range of MEC administration systems in different medical resource settings across China will maximize the generalizability of the study results.

During the first year after the trial commenced, over 300 patients have been recruited at 30 hospitals in eastern (Shanghai) and western (Sichuan province) regions of China, demonstrating feasibility of the assessment and randomization systems, use of waiver of consent or brief consent, and implementation of the treatment in ambulance settings in both high- and low-medical resources regions. There have been several protocol deviations in the ambulance: (i) eligibility deviations, mostly from including randomized patients with an onset time beyond 2 h (mostly due to wake-up stroke) or with severe comorbid disease conditions, and (ii) intervention deviations that have included not providing BP lowering to patients allocated to the intensive group or giving BP lowering to those in control group. Short transportation times, for example averages of 15 and 20 min in Shanghai and Chengdu, respectively, are the main reason why ambulance doctors have limited time to collect baseline information or deliver treatment. However, such protocol deviations improved through 2021 after further training of investigators.

The COVID-19 pandemic caused a major challenge to the recruitment, mainly by adversely influencing the behavior of patients with suspected stroke symptoms [20]. More than half of the screen failure reasons were because of a presentation time beyond the inclusion criteria of 2 hours from symptom onset. Although COVID-19 was well controlled in China by mid-2020, these late-presenting patients (or their family members) indicated that they postponed calling the emergency hotline due to concerns of “getting infected” from being exposed at a “high-risk” hospital; their first reaction was to wait and hope that the symptom(s) would mitigate or disappear. Since the recruitment schedule was slower than planned across the two initial regional centers (Shanghai and Chengdu), the TSC decided to expand the study to other regions and hospitals in 2021.

Currently, of all screened patients, the successful recruitment rate is stable at 25–30%, which equates to approximately 35 patients being randomized per month across 20 hospitals in two regions. The trial is being expanded to over 50 hospitals in 9 provinces, with the aim of recruiting to a target of 70–80 patients per month to ensure the required sample size is achieved within the proposed study period.

As the largest pre-hospital clinical stroke trial, INTERACT4 aims to establish a widely applicable treatment strategy, facilitate capacity building of stroke care in the ambulance setting, and provide reliable evidence and improve medical emergency systems.

## Trial status

The study has been approved by relevant ethics committees and regulatory bodies at country-level, local MECs and hospitals in China.

Patient enrolment commenced in March 2020 and is planned to end in December 2023. As of 31 March 2021, 377 participants have been enrolled at 30 sites in Shanghai and Sichuan province of China. The study plan to expand to more than 50 sites in 9 provinces across China during mid-2021; currently, 50 local site EC approvals have been obtained. The current protocol is version 2.0, and all protocol updates have been approved by TSC, EC, and communicated with investigators and DSMB members.

## Supplementary Information

**Additional file 1..** List of INTERACT4 collaborators

**Additional file 2..** DSMB Charter

**Additional file 3..** TSC Charter

**Additional file 4..** Informed consent materials

**Additional file 5..**

## Abbreviations

AIS: Acute ischemic stroke
BP: Blood pressure
CCC: Central Coordinating Center
CRF: Case report form
CT: Computerized tomography
DSMB: Data and Safety Monitoring Board
EC: Ethics committee
IAC: International Advisory Committee
INTERACT2: The second phase, INTEnsive blood pressure Reduction in Acute Cerebral hemorrhage Trial
INTERACT4: INTEnsive ambulance-delivered blood pressure Reduction in hyper-Acute stroke Trial
IRB: Institutional review board
MEC: Medical emergency center
mRS: Modified Rankin scale
NIHSS: National Institute of Health stroke scale
RCC: Regional Coordinating Center
PISCF: Patient information sheet and consent form
SAE: Serious adverse event
SAP: Statistical analysis plan
TGI: The George Institute for Global Health
TSC: Trial Steering Committee

## References

[1] Roth GA, Mensah GA, Johnson CO (2020). et al; GBD-NHLBI-JACC Global Burden of Cardiovascular Diseases Writing Group. Global burden of cardiovascular diseases and risk factors, 1990-2019: update from the GBD 2019 study. J Am Coll Cardiol.

[2] Wardlaw JM, Murray V, Berge E, del Zoppo G, Sandercock P, Lindley RL, Cohen G (2012). Recombinant tissue plasminogen activator for acute ischaemic stroke: an updated systematic review and meta-analysis. Lancet.

[3] Goyal M, Menon BK, van Zwam WH, Dippel DWJ, Mitchell PJ, Demchuk AM, Dávalos A, Majoie CBLM, van der Lugt A, de Miquel MA, Donnan GA, Roos YBWEM, Bonafe A, Jahan R, Diener HC, van den Berg LA, Levy EI, Berkhemer OA, Pereira VM, Rempel J, Millán M, Davis SM, Roy D, Thornton J, Román LS, Ribó M, Beumer D, Stouch B, Brown S, Campbell BCV, van Oostenbrugge RJ, Saver JL, Hill MD, Jovin TG (2016). Endovascular thrombectomy after large-vessel ischaemic stroke: a meta-analysis of individual patient data from five randomised trials. Lancet.

[4] Qureshi AI, Ezzeddine MA, Nasar A, Suri MFK, Kirmani JF, Hussein HM, Divani AA, Reddi AS (2007). Prevalence of elevated blood pressure in 563,704 adult patients with stroke presenting to the ED in the United States. Am J Em Med.

[5] Geeganage CM, Bath PM (2009). Relationship between therapeutic changes in blood pressure and outcomes in acute stroke: a metaregression. Hypertension.

[6] Anderson CS, Heeley E, Huang Y, Wang J, Stapf C, Delcourt C, Lindley R, Robinson T, Lavados P, Neal B, Hata J, Arima H, Parsons M, Li Y, Wang J, Heritier S, Li Q, Woodward M, Simes RJ, Davis SM, Chalmers J (2013). Rapid blood-pressure lowering in patients with acute intracerebral hemorrhage. New Engl J Med.

[7] Qureshi AI, Palesch YY, Barsan WG, Hanley DF, Hsu CY, Martin RL, Moy CS, Silbergleit R, Steiner T, Suarez JI, Toyoda K, Wang Y, Yamamoto H, Yoon BW, ATACH-2 Trial Investigators and the Neurological Emergency Treatment Trials Network (2016). Intensive blood-pressure lowering in patients with acute cerebral hemorrhage. New Engl J Med.

[8] Moullaali T, Wang X, Martin RH, Shipe VB, Robinson TG, Chalmers J, Suarez JI, Qureshi AI, Palesch YY, Anderson CS (2019). Blood pressure control and clinical outcomes in acute intracerebral haemorrhage: a preplanned pooled analysis of individual participant data. Lancet Neurol.

[9] Anderson CS, Huang Y, Lindley RI, Chen X, Arima H, Chen G, Li Q, Billot L, Delcourt C, Bath PM, Broderick JP, Demchuk AM, Donnan GA, Durham AC, Lavados PM, Lee TH, Levi C, Martins SO, Olavarria VV, Pandian JD, Parsons MW, Pontes-Neto OM, Ricci S, Sato S, Sharma VK, Silva F, Song L, Thang NH, Wardlaw JM, Wang JG, Wang X, Woodward M, Chalmers J, Robinson TG, Anderson CS, Huang Y, Lindley RI, Chen X, Arima H, Chen G, Li Q, Billot L, Delcourt C, Bath PM, Broderick JP, Demchuk AM, Donnan GA, Durham AC, Lavados PM, Lee TH, Levi C, Martins SO, Olavarria VV, Pandian JD, Parsons MW, Pontes-Neto OM, Ricci S, Sato S, Sharma VK, Silva F, Song L, Thang NH, Wardlaw JM, Wang JG, Wang X, Woodward M, Chalmers J, Robinson TG, Kim JS, Stapf C, Simes RJ, Hankey GJ, Sandercock P, Bousser MG, Wong KSL, Scaria A, Hirakawa Y, Moullaali TJ, Carcel C, Gordon P, Fuentes-Patarroyo SX, Benito D, Chen R, Cao Y, Kunchok A, Winters S, Coutts S, Yoshimura S, You S, Yang J, Wu G, Zhang S, Manning L, Mistri A, Haunton V, Minhas J, Malavera A, Lim J, Liu L, Kumar NN, Tay N, Jenson K, Richtering S, Tucker S, Knight E, Ivanova E, Thembani E, Odgers E, Sanders E, Small S, Vaghasiya R, Armenis M, Donnelly P, Baig MA, Blacklock N, Naidu B, Monaghan H, Smith P, Glass P, Bai X, Li Q, Zhu P, Kong L, He R, Zhao H, Lv J, Jia H, Xi Z, Cong Y, Cui B, Deng H, Guo Y, He L, Jia R, Li N, Li W, Liu M, Zhang M, Xu Z, Zhang T, Zhao Y, Gregory P, in Y, Kim SJ, Ahn JE, Kim SH, Hong YL, González-McCawley F, Martins MCO, Portales B, Wang CY, Ryu SJ, Aujla H, Lewin S, Kumar T, Barrows S, Ebraimo A, Uyen HH, Giang NA, Linh LTM, An LTT, Phuong DM, Ngoc PVB, Hang NM, Tran NTB, Hien HTT, Yen MB, Tram NTB, Truc TTT, Hoa NA, Thuan NTB, Oanh HTK, Arora D, Verma SJ, Krause M, Priglinger M, Day S, Jala S, Davies L, Ray E, Celestino S, Law LY, Wijeratne T, Ng G, Nagao K, Weiss G, Titton N, Batista C, Zãn D, Carbonera L, Ferreira K, Castro R, Martins Filho RK, Carvalho M, Libardi M, Martins G, Fagundes D, Baron G, Boehringer A, Barbosa J, Bazan R, Braga G, Luvizutto G, Ribeiro P, Winckler F, Moro C, Longo A, Liberato R, Barbosa R, Magalhães P, Portal M, Martin K, Souza A, Cuervo D, Perin D, Marques L, Oliveira F, Battaglini M, Lourenço F, Ferreira K, Silva G, Duarte L, Alves M, Sousa J, Uhehara M, Brunser A, Mazzón E, Spencer M, Acosta I, Rojo A, Rivas R, Klapp C, Carvallo L, Carvallo P, Mansilla E, Flores J, Alvarado M, Herrera A, Reyes C, Jurado F, Bustamante G, Bravo L, Matamala JM, Guerrero R, Zhou S, Ping L, Liu W, Liu L, Tian Y, Xu H, Wang J, Wang L, Zhen Z, Wang L, Zhang J, Yan M, Wang L, Zhang Q, Tao X, Liu C, Shi J, Zhang X, Tai L, Xu L, Lu H, Nie H, Li X, Zhou J, Liu Y, Gong P, Tian Y, Zhao H, Zhang J, Li R, Wang X, Chen Q, Li Y, Wu L, Zhang J, Jia L, Guo X, Li X, Chen G, Lin B, Zhu W, Yang K, Zhang J, Zhang Z, Xie C, Wu D, Zhang Z, Li X, Wang Y, Liu D, Liu Z, Liang L, Cao Q, Zhang X, Xia J, Li X, Weng Y, Li J, Xu T, Geng D, Yan X, Wang D, Zhao N, Li J, Wang D, Tang Z, Wang L, Yin W, Wang S, Wang D, Huang W, Yang Y, Song A, Hao Y, Zhang A, Qiao B, Yang J, Yan H, Wei X, Tao Z, Liu H, Lv Y, Yang H, Han L, Mao X, Ge L, Zhang Y, He S, Zhang Q, Zhao H, Jiang J, Yan M, Liu D, Wu W, Wang H, Wang Y, Yang L, Tang Y, Sun H, Li F, Li G, Sun Y, Zhang H, Wu Y, Huang L, Geng C, Jin Z, Zhu J, Zhang F, Zhang Y, Zhang Z, Zheng R, Shen H, Liu F, Chen C, Li G, Chen S, Zhou L, Hu B, Zou Z, Liu J, Zhang X, Chang X, Wang D, Zhang S, Huang Q, Liu X, Liu S, He W, Feng J, Li L, Chen X, Zhuang X, Liu Y, Zheng W, Lai Y, Zhou Y, Duan H, Cao Q, Yang Q, du J, Lin Q, Xu E, Zhan L, Yang L, Huang Q, Wu J, Feng X, Wei C, He J, Wang B, Liu X, Li W, Chen P, Guo F, Dai H, Dai M, Zeng X, Wang D, Chen B, Long F, Su Q, Wang Y, Bao B, Wu T, Wu X, Shao Y, Nie H, Zhang X, Li S, Xu Y, Castellanos JA, Muñoz-Collazos M, Solano E, Leung WHT, Sureshbabu S, Sharma SN, George S, Shekhar S, Singla S, Saini L, Sunita, Kate M, Sarvotham R, William AG, Deepak A, BK M, Benny R, Bolegave V, Basle M, Gore S, George P, Kumaravelu S, Rahamath S, Raj PG, Devi AR, Sharma A, Prajapati J, Parmar M, Patel D, Panchal T, Gorthi SP, Prabhu V, Prabhu A, Chandran V, Chatterjee A, Nair R, Nambiar VK, TS D, TP S, Ajai V, Paul S, Natarajan PC, Chittibabu D, Borah NC, Ghose M, Choudhury N, Gohain P, Kalita K, Duberkar D, Pawar N, Bhaviskar R, Caterbi E, Cenciarelli S, Condurso R, Gallinella E, Greco L, Marando C, Mastrocola S, Mattioni A, Sacchini E, Sicilia I, Gallina A, Giannandrea D, Marsili E, Mazzoli T, Padiglioni C, Corea F, Guidubaldi A, Micheli S, Barbi M, Kim J, Song HJ, Jeong HS, Lim JG, Park SM, Lee KB, Hwang HW, Kwon SU, Kang DW, Kim YJ, Kim BJ, Park JM, Kang K, Kim B, Kwon O, Kim YW, Lee JJ, Hwang YH, Kwon HS, Koo J, Lee K, Kim T, Ahn A, Rha JH, Park HK, Yoon CW, Chan B, Teoh HL, Paliwal P, Wong LYJ, Chen JT, de Silva DA, Chang HM, Fabiaña N, Marti J, Delgado R, Martínez A, Prats L, Camps P, Liou CW, Tan TY, Liu CF, Cheng HH, Po HL, Lin YJ, Chou CL, Lin CH, Yen CC, Chang YT, Hsu YT, Lee JD, Lee M, Huang YC, Wu CY, Huang YC, Suwanwela NC, Chutinet A, Likitjaroen Y, Roongpiboonsopit D, Charnwut S, Dyker A, Hossain M, Muddegowda GK, Sanyal R, Roffe C, Natarajan I, Finney K, Sztriha L, Teo J, Chan FK, Lim J, Chitando B, Clarke B, Patel B, Khan U, Ghatala R, Trippier S, Kalra L, Manawadu D, Sikondari N, Aeron-Thomas J, Sunman W, Wilkes G, Richardson C, Buch A, Jackson B, Halse O, Mashate S, Wilding P, Nguyen V, Qadiri MR, Rashed K, Board S, Buckley C, Smith C, James M, Keenan S, Bouring A, England T, Donnelly R, Scott J, Maddula M, Beavan J, Perry R, Francia N, Watchhurst C, Banaras A, Ashton A, Mistri A, Musarrat K, Eveson D, Kallingal J, Perez J, Harrison L, Marsden T, Furnace J, Clarke R, Reid J, Warburton E, Macleod MJ, Mitchell J, Day D, Church N, Amis E, Price C, Rodgers H, Whiting R, Hussain M, Harvey M, Brown S, Foot J, Tryambake D, Broughton D, Bergin A, Annamalai A, Dixon L, Weir N, Blank C, Harkness K, Ali A, Richards E, Stocks K, Bruce DW, Wani M, Anjum T, Krishnan M, Nguyen Huy T, le Tuan AT, Cam LDT, Kim TNT, Nguyen BP, Dat AN, van CN, Duy TM, Viet PD, Tien DN, van TV, le Kim K, Ngoc TB, le Thanh TT, Hoanh SN, Phuoc SP, van TT, Thi BD, Thu HNT, Duy MN, van DN (2019). Intensive blood pressure reduction with intravenous thrombolysis therapy for acute ischaemic stroke (ENCHANTED): an international, randomised, open-label, blinded-endpoint, phase 3 trial. Lancet.

[10] Anadani M, de Havenon A, Mistry E, Anderson CS (2021). Blood pressure management after endovascular therapy: an ongoing debate. Stroke.

[11] RIGHT-2 Investigators (2019). Prehospital transdermal glyceryl trinitrate in patients with ultra-acute presumed stroke (RIGHT-2): an ambulance-based, randomised, sham-controlled, blinded, phase 3 trial. Lancet.

[12] Bath PM, Woodhouse LJ, Krishnan K, Appleton JP, Anderson CS, Berge E, Cala L, Dixon M, England TJ, Godolphin PJ, Hepburn T (2019). Prehospital transdermal glyceryl trinitrate for ultra-acute intracerebral hemorrhage: data from the RIGHT-2 trial. Stroke.

[13] Delcourt C, Huang Y, Wang J, Heeley E, Lindley R, Stapf C, Tzourio C, Arima H, Parsons M, Sun J, Neal B, Chalmers J, Anderson C, INTERACT2 Investigators (2010). The second (main) phase of an open, randomised, multicentre study to investigate the effectiveness of an intensive blood pressure reduction in acute cerebral haemorrhage trial (INTERACT2). Int J Stroke.

[14] Saver JL (2011). Optimal end points for acute stroke therapy trials: best ways to measure treatment effects of drugs and devices. Stroke.

[15] Brott T, Adams HP, Olinger CP, Marler JR, Barsan WG, Biller J, Spilker J, Holleran R, Eberle R, Hertzberg V (1989). Measurements of acute cerebral infarction: a clinical examination scale. Stroke.

[16] Rabin R, de Charro F (2001). EQ-5D: a measure of health status from the EuroQoL group. Ann Med.

[17] Saver JL, Starkman S, Eckstein M, FAST-MAG Investigators and Coordinators (2015). Prehospital use of magnesium sulfate as neuroprotection in acute stroke. N Engl J Med.

[18] Haybittle JL (1971). Repeated assessment of results in clinical trials of cancer treatment. Br J Radiol.

[19] Moore GF, Audrey S, Barker M, Bond L, Bonell C, Hardeman W, Moore L, O'Cathain A, Tinati T, Wight D, Baird J (2015). Process evaluation of complex interventions: Medical Research Council guidance. BMJ.

[20] Song L, Ouyang M, Sun L, Chen C, Anderson CS (2020). Impact of COVID-19 on patient behavior to stroke symptoms in China. Cerebrovasc Dis.

