# Supplementary material for: INTEnsive ambulance-delivered blood pressure Reduction in hyper-ACute stroke Trial (INTERACT4): study protocol for a randomized controlled trial
Source: Trials. 2021 Dec 6;22:885. doi: 10.1186/s13063-021-05860-y (PMC8646007; doi:10.1186/s13063-021-05860-y)
Supplement: Supplementary file 1 — Additional file 1.. List of INTERACT4 collaborators [file 13063_2021_5860_MOESM1_ESM.pdf]

List of INTERACT4 Collaborators

The INTERACT4 investigators and collaborators are as follow:

Trial Steering Committee: C.S. Anderson (Chair & Principal Investigator), G. Li (Co-Principal Investigator), J. Yang (Co-Principal Investigator), L. Song (Co-Principal Investigator), H. Arima

International Advisory Committee: P. Bath, T. Robinson, G. Ford, N. Sprigg, E. Sandset, J. Saver, B. Worp, Z. Liu

Data Safety and Monitoring Committee: J. Dawson (Chair), L. Wong; B. Peng

Trial Statistician: L. Billot, Q. Li

Data Management: Y. Ning

Central Coordinating Centre: ***The George Institute China* -** L. Song, X. Chen, Z. Yang, W. Yu

Regional Coordinating Centres: ***Shanghai East Hospital*** - G. Li, C. Chen, F. Liu; ***The First Affiliated Hospital of Chengdu Medical College*** - J. Yang, Y. Guo, Y. Lin, R. Hu, H. Cheng, W. Ma, K. Liao

Site Principal Investigators - *Shanghai Pudong New Area Medical Emergency Centre*: C. Zhang; *Xuzhou Medical Emergency Centre:* H. Jin; *Shanghai East Hospital*: G. Li; *The First Affiliated Hospital of Chengdu Medical College*: J. Yang; *Zigong Fourth People's Hospital:* P. Xu; *Guanghan People's Hospital:* X. Wu; *Shanghai Seventh People's Hospital:* F. Wang; *Fushun People's Hospital:* L. Wu; *People's Hospital of Deyang City:* C. Wang; *Qianwei People's Hospital:* Y. Peng; *Shanghai Pudong New District People's Hospital:* X. Zhao; *Shanghai Pudong New District Zhoupu Hospital:* X. Xu; *The Second Affiliated Hospital of Chengdu Medical College:* X. Liu; *Chengfei Hospital:* X. Song; *Dazhu Hospital:* Z. Li; *Jiangyou People's Hospital:* H. Zhang; *Sichuan Tianfu New Area People's Hospital:* D. Yu; *Shanghai Pudong New District Punan Hospital:* Y. Wang; *The People's Hospital of Jianyang City:* X. Tang; *Yanting People's Hospital:* H. Liu; *Xindu Hospital of Traditional Chinese Medicine:* X. Cao; *Yibin NO.4 People's Hospital:* Y. Liu; *The People's Hospital of Zhongjiang:* Y. Duan; *The Third People's Hospital of Chengdu:* H. Liu; *Jiangyou 903 Hospital:* C. Li; *The Second People's Hospital of Neijiang:* J. Huang; *The First People's Hospital of Yibin:* H. Li; *Xichang People's Hospital:* C. Fan; *Mianyang Central Hospital:* Y. Tang; *Nanchong Central Hospital:* Y. Ji; *The Sixth People's Hospital of Chengdu:* G. Li; *The First People's Hospital of Shuangliu District, Chengdu:* Y. Huang; *Xuzhou Central Hospital:* G. Chen; *Ya'an People's Hospital:* J. Wang.
