# Supplementary material for: INTEnsive ambulance-delivered blood pressure Reduction in hyper-ACute stroke Trial (INTERACT4): study protocol for a randomized controlled trial
Source: Trials. 2021 Dec 6;22:885. doi: 10.1186/s13063-021-05860-y (PMC8646007; doi:10.1186/s13063-021-05860-y)
Supplement: Supplementary file 2 — Additional file 2.. DSMB Charter [file 13063_2021_5860_MOESM2_ESM.pdf]

INTEnsive ambulance-delivered blood pressure Reduction in hyper-Acute stroke Trial

Data Safety Monitoring Board Charter

Co-Principal Investigator: Professor Craig Anderson, The George Institute for Global Health, China

Co-Principal Investigator: Professor Gang Li, Shanghai East Hospital, Tongji University, China

Co-Principal Investigator： Professor Jie Yang, The First Affiliated Hospital of Chengdu Medical College, China

Co-Principal Investigator: Dr Lili Song , The George Institute for Global Health, China

ClinicalTrials.gov: NCT03790800

Chinese Trial Registry: ChiCTR1900020534

Trial Sponsor: The George Institute for Global Health, China

Shanghai East Hospital, Tongji University, China

The First Affiliated Hospital of Chengdu Medical College, China

Date of Document:

Version 1.0, 6 Nov 2019

DSMB Member Statement & Signature Page

Members of the Data and Safety Monitoring Board (DSMB) undertake their respective roles on the DSMB in a volunteer capacity. Each member is obliged to comply with the requirements set forth within this Charter.

It is the policy of the DSMB that members shall keep all information relating to the INTERACT4 study completely confidential unless compelled by legal process to disclose such information, or as otherwise agreed by the DSMB. While Board members are free to discuss the result of Board action items, disclosing any information concerning the discussion of such items during the Board meeting is prohibited. Board members acknowledge that any violation of this policy could cause harm to the INTERACT4 trial and discredit the study.

By signing below, all members agree to the above statements and in doing so shall be indemnified by The George Institute.

Name	Signature	Date
**Professor Jesse Dawson**	[signature]	6th November 2019
**Professor Lawrence Wong**	[signature]	6th November 2019
**Professor Bin Peng**	[signature]	6th November 2019

Table of Contents

DSMB Member Statement & Signature Page 2
1 Introduction 4
2 Objectives of the DSMB 4
3 Membership 5
3.1 Member Selection 5
3.2 DSMB Members 5
3.3 DSMB Statisticians (Non-DSMB Members) 5
3.4 Membership 6
3.5 Conflicts of Interest 6
4 Roles & Responsibilities 6
4.1 DMSB Chairperson 6
4.2 Members 7
4.3 Statistics Group 7
4.4 Steering Committee 8
4.5 Project Manager 8
5 Preparation & Distribution of Data for Review 9
6 Meetings & Sessions 9
6.1 Meeting Overview 9
6.2 Meeting Schedule 10
6.3 Meeting Format 11
6.4 Open Sessions 11
6.5 Closed Sessions 11
6.6 Meeting Reports, Statements & Minutes 11
6.6.1 Open Reports 12
6.6.2 Closed Reports 12
6.6.3 DSMB Statements and Minutes 12
7 Statistical Monitoring Guidelines 13
8 Stopping the Trial 14
9 Maintenance of Documentation 14
10 Lines of Communication 15
11 Confidentiality 15
12 Reference 15

1 Introduction

INTERACT4 is a multicentre, ambulance-delivered, prospective, randomized, open-label, blinded endpoint (PROBE) study to assess the effects of hyperacute intensive blood pressure (BP) lowering initiated in ambulance setting on (i) functional outcome in patients with acute stroke (ii) safety in patients with confirmed acute stroke and other conditions that were initially suspected as acute stroke (i.e. stroke mimic).

This Charter will define the primary responsibilities of the Data Safety Management Board (DSMB), its relationship with other trial committees, its membership, and the purpose and timing of its meetings. The Charter will also provide the procedures for ensuring confidentiality and proper communication, the statistical monitoring guidelines to be implemented by the DSMB, and an outline of the content of the Open and Closed Reports that will be provided to the DSMB.

2 Objectives of the DSMB

The DSMB will consist of physicians experienced in clinical studies. The committee will be supported by 2 statisticians from the George Institute for Global Health, one is unblinded and the other is blinded. The independent DSMB will review safety data on an ongoing basis and may recommend the INTERACT4 Steering Committee stop or amend the study based on safety findings.

Furthermore, the DSMB with be responsible for:

- Safeguarding the interests of trial participants;
- Reviewing the research protocols and plans for data and safety monitoring;
- Reviewing data monitoring reports provided by the study statistician;
- Reviewing the progress of the study and monitoring adherence to the protocol, participant recruitment, outcomes, data quality, complications, and other issues related to participant safety;
- Monitoring the assumptions underlying sample size calculations for the study (Steering Committee and Operations Committee should be kept blinded) and alert the investigators if they see substantial departures as the data accumulate;
- Ensuring the confidentiality of the study data and the results of monitoring;
- Assessing the safety and efficacy of the interventions during the trial;
- Monitoring the overall conduct of the clinical trial;
- Providing recommendations about stopping or continuing the trial to the trial Steering Committee;
- Contributing to enhancing the integrity of the trial;
- Formulating recommendations in relation to the selection, recruitment, or retention of participants, or their management, or to improving their adherence to protocol-specified

regimens and retention of participants, and the procedures for data management and quality control.

The DSMB will be advisory to the Steering Committee. The Steering Committee will be responsible for promptly reviewing the DSMB recommendations, to decide whether to continue or terminate the trial, and to determine whether amendments to the protocol or changes in study conduct are required.

3 Membership

3.1 Member Selection

The DSMB is an independent, multidisciplinary group including experts in clinical medicine, neurology, stroke and statistics and epidemiology. All members have experience in the conduct and monitoring of randomised control trials

3.2 DSMB Members

**Professor Jesse Dawson (Chair)**	University of Glasgow ,Scotland, UK<br>E: Jesse.Dawson@glasgow.ac.uk<br>T: +44 0141 4515868
**Professor Lawrence Wong**	The Chinese University of Hong Kong ,Hong Kong, China<br>E: ks-wong@cuhk.edu.hk<br>T: +85 2 26323144/90177461
**Professor Bin Peng**	Peking Union Medical College Hospital, Beijing, China<br>E: pengbin3@hotmail.com<br>T: +86 010 69156371

3.3 DSMB Statisticians (Non-DSMB Members)

Two independent statisticians of The George Institute, one blinded and one unblinded, will be assigned to the DSMB to facilitate data presentation and analyses. These statisticians are not DSMB members and will have no voting rights for any decisions to be made.

**Laurent Billot (Blinded Statistician)**	Statistics Division<br>The George Institute for Global Health, Sydney, Australia<br>T: +61 2 80524581/+33 7 60982300<br>Mob: +61 414 571 031<br>E: lbillot@georgeinstitute.org
**Qiang Li (Unblinded Statistician)**	Statistics Division<br>The George Institute for Global Health, Sydney, Australia

T: +61 2 80524516
Mob: +61 412 761 511
E: qli@georgeinstitute.org

3.4 Membership

DSMB membership is to be for the duration of the clinical trial. If any member leaves the DSMB during the course of the trial, the Steering Committee and/or investigators will promptly appoint their replacements.

The quorum of the DSMB is validly constituted by the Chair plus the other 2 DSMB members. Any queries can be discussed via email or a separate special meeting.

The DSMB may call upon other experts to attend DSMB meetings to provide information and/or advice regarding unanticipated findings or issues. These individuals are not considered DSMB members and cannot vote in DSMB meetings.

3.5 Conflicts of Interest

DSMB membership is restricted to individuals free of relevant significant conflicts of interest. The source of these conflicts may be financial, scientific or regulatory in nature. Individuals who fulfil any of the following criteria are automatically unqualified from membership:

- Study investigators
- Individuals employed by The George Institute, Shanghai East Hospital, The First Affiliated Hospital of Chengdu Medical College
- Individuals employed by any company that makes or sells any of the blood pressure lowering agents or any other agents used in relation to the treatment of stroke.

The DSMB members will disclose to fellow members any potential conflicts of interest, and the DSMB will be responsible for deciding whether any materially impact on their objectivity.

4 Roles & Responsibilities

4.1 DMSB Chairperson

The INTERACT4 Steering Committee has appointed Jesse Dawson as the Chairperson of the DSMB. The role of the Chairperson includes:

- Signing off on the DSMB charter (and any subsequent amendments), indicating the agreement of the DSMB to conduct its operations in accordance with the charter
- Ensuring that DSMB meetings are scheduled
- Working with the Statistics Group to ensure that the Closed DSMB Report, consisting of unblinded data listings and summaries, is received by the DSMB members only within the given time frames
- Chairing the DSMB meetings

- Acting as the contact between the DSMB and the Steering Committee by discussing the issues and representing the views of the DSMB without jeopardising the integrity of the data
- Signing the DSMB meeting minutes and the DSMB Meeting Reports summarising the conclusions and recommendations of the DSMB from each meeting
- Informing the Steering Committee Chairperson of the need for additional DSMB meetings, proposed meeting date(s), specifications for data review and any identified problems

The DSMB Chairperson will receive administrative support from the Statistics Group as required.

4.2 Members

Each member is responsible for maintaining strict confidentiality of the study data. Members will not share any study data or information about the study with any individual external to the DSMB. The blinded statistician may contact the unblinded statistician in the Statistics Group directly with questions regarding the operational details associated with the data analyses and summary tables.

Each member will review the Data Monitoring Report thoroughly prior to each DSMB meeting. A member who believes he or she may have a potential intellectual or financial conflict of interest during the course of review of the data must inform the Chairperson of the DSMB. In such cases, the DSMB meeting minutes must document the disclosure of the potential conflict of interest and the outcome of the discussion, e.g. abstention of member from voting.

4.3 Statistics Group

The Statistics Group is based in The George Institute for Global Health in Sydney, Australia. Their names, roles in the project, and contact information are included in Section 3.3. The Statistics Group will have primary responsibility for:

- Ensuring that the Data Monitoring Report provided to the DSMB is complete and accurate
- Storing copies of the Data Monitoring Reports until after the completion of the INTERACT4 study and database lock
- If requested, after database lock sending to the Steering Committee a copy of each Data Monitoring Report along with any other applicable documentation
- Performing additional analyses that are requested by the DSMB, which may have the potential to unblind individuals to the results of the study. All such additional analyses will be similarly archived and made available at study termination.

In addition, the Statistics Group will assist the DSMB Chairperson with the following responsibilities:

- Oversee the preparation of the Closed DSMB Report, ensuring that it includes the required unblinded data listings and summaries, and that it is received by the DSMB members within the given time frames
- Record and finalise minutes of Closed Sessions, review and help finalise Open Session minutes prepared by the INTERACT4 team.

- Ensure that the DSMB meeting minutes from the Closed Sessions and other documentation are maintained appropriately.

4.4 Steering Committee

The Steering Committee is a multidisciplinary group, collectively, have the scientific, medical and clinical trial management experience to conduct and evaluate the trial, they are convened to review the recommendations of the DSMB. The Steering Committee is also responsible for the design, conduct and analysis of the clinical trial.

The Steering Committee is jointly responsible with the DSMB for safeguarding the interests of participating patients and for the conduct of the trial. Recommendations to amend the protocol or conduct of the study made by the DSMB will be considered and accepted or rejected by the Steering Committee. Furthermore, the Steering Committee will be responsible for deciding whether to continue or to stop the trial based on the DSMB recommendations. If the DSMB statisticians feel conflicted in decision making by having knowledge of the unblinded data, they will recommend an independent statistician be brought in to advise the Steering Committee.

The DSMB will be notified of all decisions made by the Steering Committee, including changes to the protocol or to study conduct. The DSMB concurrence will be sought on all substantive recommendations or changes to the protocol or study conduct prior to their implementation.

The Steering Committee will maintain confidentiality of all information it receives other than that contained in the Open Reports (Section 6.6.1) until after the trial is completed or until a decision for early termination has been made.

Furthermore, the INTERACT4 Steering Committee is responsible for:

- Constituting the DSMB
- Appointing the DSMB Chairperson
- Agreeing to the DSMB charter
- Coordinating resources and procedures to support DSMB operations
- Providing the DSMB with relevant information regarding the drug and conduct of the clinical trial including protocol amendments
- Reviewing unblinded information from the DSMB in the event that the DSMB recommends to stop the trial prior to scheduled closure

4.5 Project Manager

The Project Manager or delegate is responsible for:

- Ensuring that the DSMB charter is signed by all members of the DSMB
- Scheduling DSMB meetings
- Making the appropriate DSMB meeting arrangements; booking rooms, and circulating teleconference dial-in details
- Recording minutes for the Open Session of the DSMB meeting and obtaining approval for these from the Chair of the DSMB before circulating to all those who attended.

- Ensuring that the DSMB meeting minutes from the Open Sessions and other documentation are maintained appropriately.

5 Preparation & Distribution of Data for Review

To enhance the integrity and credibility of the trial, procedures will be implemented to ensure the DSMB has sole access to evolving information from the clinical trial regarding comparative results of efficacy and safety data, aggregated by treatment arm. An exception will be made to permit access to the unblinded statistician who will be responsible for serving as a liaison between the database and the DSMB. The trial Central Coordinating Centre (CCC) at The George Institute China will provide the Chair of the DSMB with information on any serious adverse events (SAEs) considered related to the study treatment, and will also be responsible for satisfying the standard requirements for reporting of relevant events to the regulatory authorities.

At the same time, procedures will be implemented to ensure proper communication is achieved between the DSMB, trial investigators and the sponsor. To provide a forum for exchange of information among various parties that share responsibility for the successful conduct of the trial, a format for Open Sessions and Closed Sessions will be implemented. The intent of this format is to enable the DSMB to preserve confidentiality of the comparative efficacy results while at the same time providing opportunities for interaction between the DSMB and others who have valuable insights into trial-related issues.

The INTERACT4 study database is held by Bioknow, maintained by Shanghai East Hospital, Tongji University, China and The George Institute for Global Health, China. Likewise, the randomisation codes have been prepared and are held by Bioknow. The preparation of DSMB reports will be done on the basis that only the independent Statistics Group and the DSMB will have access to unblinded data.
The unblinded statistician will obtain unblinded data extracts one month prior to the planned DSMB meeting. The data will be saved in an access-restricted folder, the unblinded statisticians are the only people with access to both the study data and the randomisation codes.

The preparation of the Data Monitoring Reports will be done to an agreed standard analysis and reporting format developed by the independent Statistics Group with the support of the project statistician at The George Institute and under the direction of the DSMB. The format will be signed off by the Steering Committee.

The independent Statistics Group will send DSMB members Data Monitoring Reports at least 5 working days prior to scheduled meetings in a password-encrypted PDF file.

6 Meetings & Sessions

6.1 Meeting Overview

An initial meeting of the DSMB will be held prior to receipt of any safety or efficacy data from INTERACT4. The purpose of the meeting will be to:

- Familiarise DSMB members with INTERACT4
- Review the DSMB charter and complete the procedural sections of the DSMB charter
- Develop more specific operational guidelines, i.e. frequency of meetings, logistics of meetings

Subsequent meetings will be scheduled at approximately 6-monthly intervals. DSMB members are expected to participate in each meeting. Meetings will be held by teleconference, or in person where feasible. On occasion, the DSMB may require consultants with additional expertise in the review of safety or efficacy data. These consultants will be bound by the same confidentiality requirements as regular DSMB members. The Steering Committee must agree to the objectives and the presence of additional participants at DSMB meetings. This information must also be documented in the DSMB meeting minutes and the DSMB Meeting Reports.

The DSMB may deem it necessary to hold additional, unscheduled, meetings. The DSMB Chairperson will ensure that the request for additional analyses and meetings are consistent with the objectives of the DSMB as outlined in the charter. The DSMB Chairperson must inform the Steering Committee Chairperson of the issues, proposed meeting date(s), and specifications for data review and obtain agreement.

Following the completion of each DSMB meeting held during the course of the trial, a recommendation will be made to the Steering Committee to continue or terminate the trial. This recommendation will be based primarily on safety and efficacy considerations and will be guided by statistical monitoring guidelines defined in this charter. Should the DSMB consider a recommendation, by any member to terminate the trial early, a full vote of the DSMB will be required. This information should be forwarded to the trial Principal Investigator as rapidly as possible.

6.2 Meeting Schedule

To ensure ongoing safety surveillance the DSMB will review unblinded data periodically. The review will be based upon the best available un-adjudicated investigator-reported data.

The committee will conduct both periodical safety reviews and formal interim analyses as follows:

- The first analysis meeting of the DSMB is planned within 6 months from the first patient enrollment of the study to review initial safety data and finalise the format of reports.
- Subsequent safety review meetings will be conducted approximately every six months in the initial stages of the trial. Depending on trial progress, the meeting interval may be extended to annually. The safety reviews will concentrate on safety assessment and will not include formal testing of the efficacy data.
- Two 'Formal Interim Analysis' meetings will be held to review data relating to treatment efficacy, patient safety and quality of trial conduct in conjunction with the following scheduled safety review meetings after 33% and 66% of randomised patients are enrolled. Formal interim reviews will be held by teleconference or face-to-face.

- The DSMB will also respond to specific requests made by the INTERACT4 Steering Committee.

6.3 Meeting Format

The DSMB meetings will begin with an Open Session followed by a Closed Session. INTERACT4 team members, including the Principal Investigator may present pertinent study information to the DSMB members during the Open Session. Investigators or experts serving as ad hoc advisors may be requested to attend an open session of the meeting. The Closed Session will be limited to the DSMB members, consultants to the DSMB if needed, and designated staff from the Statistics Group for presentation of the unblinded data. An Executive Session can be called with DSMB members only if required. INTERACT4 team members and Steering Committee members are excluded from participating in any Closed or Executive Sessions of the DSMB.

6.4 Open Sessions

In order to allow the DSMB to have adequate access to information provided by the trial investigators, or by members of the regulatory authorities, a joint session between these individuals and DSMB members (Open Session) will be held before the Closed Session. If necessary, further Open Session can be held, on request either in the middle or end of the Closed Session. Open Sessions give the DSMB an opportunity to query these individuals about issues that have arisen during their review of the previous Closed Reports. With this format, important interactions are facilitated through which problems affecting trial integrity can be identified and resolved.

An INTERACT4 coordinating centre staff member will prepare the minutes of the Open Session and distribute them to DSMB members and investigators.

6.5 Closed Sessions

Sessions involving only DSMB members and the unblinded statistician will be held to allow discussion of confidential data from the clinical trial, including information about the relative efficacy and safety of interventions. In order to ensure that the DSMB will be fully informed in its primary mission of safeguarding the interest of participating patients, the DSMB will be unblinded in its assessment of safety and efficacy data. During these sessions, the DSMB will develop a consensus on its list of recommendations, including the continuity or termination of the trial.

The minutes of the DSMB meetings Closed Session will be prepared by the unblinded statistician and distributed to DSMB members only.

6.6 Meeting Reports, Statements & Minutes

For each DSMB meeting, both safety reviews and interim analyses, Open and Closed Reports will be provided. The Open and Closed Reports should provide information that is accurate, with follow-up that is complete to within approximately one month of the date of the DSMB meeting. The Reports should be provided to DSMB members approximately two weeks prior to the date of the meeting. The unblinded statistician(s) from The George Institute will prepare both the Open and Closed Reports.

6.6.1 Open Reports

Open Reports, available to all who attend the DSMB meetings, will include:

- Data on recruitment
- Study accrual by month and by site, including an assessment of whether recruitment targets are being met
- Pooled data on eligibility violations
- Major protocol changes
- BP measurement at time points after randomisation;
- The rate of achieving the target of BP within 30 minutes after randomisation in intervention group.
- Time between randomisation and achievement of BP target by intervention group.
- Baseline characteristics (pooled by treatment regimen)
- Completeness of follow-up and compliance
- SAE (including death) summary and detailed list
- Pooled analysis of primary outcome
- Details of the number of pending and missing case report forms (including the number of seriously overdue follow-ups), and the number with outstanding data items.
- Any other relevant information (such as updated Cochrane reviews).

6.6.2 Closed Reports

Closed Reports, available only to those attending the Closed Sessions of the DSMB meeting, will include:

- Analyses of primary and secondary efficacy endpoints (for interim reviews)
- Subgroup and baseline-adjusted analyses
- Analyses of serious adverse events
- Open Report analyses that are displayed by intervention group

The reports for the safety reviews will be a subset of the reports prepared for the formal interim analyses.

6.6.3 DSMB Statements and Minutes

DSMB Meeting Statements summarising the conclusions and recommendations of the DSMB will be drafted after each meeting. The DSMB Chairperson will oversee the finalisation of the meetings' minutes and statements and sign both documents. The DSMB meeting minutes should include important considerations that led to the DSMB recommendations. Closed Session minutes will not be sent to the Steering Committee until after the completion of the study and database lock. The

DSMB statement, which will be sent to the Steering Committee, will include DSMB conclusions and recommendations without reference to unblinded data. The Chair of the Steering Committee will provide the statement to the Project Manager for Human Research Ethics Committee (HREC) reporting and sending to participating investigators.

7 Statistical Monitoring Guidelines

The INTERACT4 DSMB will monitor safety data on an ongoing basis.

During the period of recruitment into the study, interim analyses of the proportion of patients alive and independent, or dead (at hospital discharge and at 3 months), or with other major outcome events will be supplied, in strictest confidence, to all members of the DSMB, along with any other analyses that the DSMB may request.

Considering these analyses, the DSMB will advise the Principal Investigator if, in their view, the randomised comparisons have provided both:

- ‘Proof beyond reasonable doubt' that for all, or some, a treatment is clearly indicated or clearly contra-indicated and,
- Evidence that might reasonably be expected to lead many clinicians conversant with the available evidence to materially change their practice regarding blood pressure lowering in suspected stroke patients in ambulance.

The DSMB will base the primary review on the entire randomised trial population although additional analyses of subgroups may be done as requested by the DSMB.

A recommendation to discontinue INTERACT4 prematurely will be based upon there being clear evidence that the treatment provides protection or causes harm for an important clinical outcome. The final recommendation to the Steering Committee will remain at the discretion of the DSMB but will be based upon agreed standards for the interpretation of interim analyses in clinical trials. The Steering Committee will subsequently have the responsibility of evaluating and implementing as they consider appropriate the recommendations provided by the DSMB.

A recommendation to modify INTERACT4 will be accompanied by the maximum possible information that the DSMB can provide to the Steering Committee without affecting the integrity of the trial. Once again, the Steering Committee will have the responsibility of evaluating and implementing the recommendations as they consider appropriate.

If additional expert opinion is to be sought or additional analyses are required prior to making a recommendation, the DSMB will work to schedule another meeting at the earliest possible opportunity.

The decision to stop a trial temporarily or indefinitely will be considered in hand with ensuring safety for the trial participants and the impact premature termination will have on clinical practice. The Haybittle-Peto rule[1] will be used as a guide for proof beyond reasonable doubt in the monitoring of both efficacy and safety information in the trial.

The DSMB will work on the principle that a difference of at least 3 standard errors in an interim analysis of a major outcome event (e.g. shift in death and disability according to full range scores on mRS in stroke patients at 90 days) between patients allocated to the intensive or the control group, to justify halting or modifying the study before the planned completion of recruitment. This criterion (Peto rule) has the practical advantage that the exact number of interim analyses is of less importance, and so no fixed schedule is proposed.[1]

8 Stopping the Trial

Following each DSMB Meeting, the board will recommend to the Steering Committee one of the following options in the DSMB statement:

- Modification of the study
- Termination of the study early –
    - Clear and substantial evidence of benefit;
    - Data suggests the risk of adverse events substantially outweighs the potential benefits
- Continue the study unchanged
- Additional expert review after which a recommendation will be made
- Additional analyses by the Statistics Group after which a recommendation will be made

9 Maintenance of Documentation

The DSMB Chairperson with the support of the Statistical Group and Project Manager will compile and maintain the following documents:

- Copy of the charter
    - Charter amendments
    - Associated attachments and addenda
- Protocols and protocol amendments for the INTERACT4
- Curriculum vitae for each DSMB member
- Copies of the Closed DSMB Reports provided only to the DSMB members
- DSMB Meeting Minutes
    - Including conclusions or recommendations concerning the conduct or evaluation of the trial and any important considerations that led to the conclusions and recommendations
- DSMB Statements
- Copies of key correspondence related to the DSMB

Upon completion of the trial and closure of the relevant clinical database(s), the documents will be forwarded to the Steering Committee for archiving.

10 Lines of Communication

All communication from the DSMB will be through the DSMB Chairperson to the Steering Committee Chairperson and then communicated to Steering Committee members.

The DSMB Chairperson will send to the Steering Committee Chairperson a copy of the Open DSMB Report within 5 working days of each meeting, containing the committees' recommendations, thereby documenting that the DSMB has reviewed the data. The report will divulge no details of DSMB discussions and especially no information regarding unblinded data. The Steering Committee Chairperson will inform the DSMB Chairperson of any decisions made regarding changes to the conduct of the trial within 5 days of receiving the DSMB Meeting Report.

11 Confidentiality

All materials, discussions and proceedings of the DSMB are completely confidential. DSMB members and other participants in DSMB meetings (such as the unblinded statisticians) are expected to maintain confidentiality, and will refrain from revealing to the trial Steering Committee, or any other party, information that would lead to compromising the integrity of the trial unless such release is required to protect patient safety.

12 Reference

1. Haybittle JL. Repeated assessment of results in clinical trials of cancer treatment. Br J Radiol 1971; 44:793-97
