# Supplementary material for: INTEnsive ambulance-delivered blood pressure Reduction in hyper-ACute stroke Trial (INTERACT4): study protocol for a randomized controlled trial
Source: Trials. 2021 Dec 6;22:885. doi: 10.1186/s13063-021-05860-y (PMC8646007; doi:10.1186/s13063-021-05860-y)
Supplement: Supplementary file 3 — Additional file 3.. TSC Charter [file 13063_2021_5860_MOESM3_ESM.pdf]

INTEnsive ambulance-delivered *blood pressure* Reduction in hyper-Acute stroke Trial

STEERING COMMITTEE CHARTER

Chair, Independent Steering Committee: Professor Craig Anderson (The George Institute for Global Health, Beijing Representative Office, China; Faculty of Medicine, University of New South Wales, Australia)

Principal Investigator: Professor Craig Anderson (The George Institute for Global Health, Beijing Representative Office, China; Faculty of Medicine, University of New South Wales, Australia)

Co-Principal Investigator: Professor Gang Li (Shanghai East Hospital, Tongji University, China),professor Jie Yang （The First Affiliated Hospital of Chengdu Medical College, China),Dr Lili Song (The George Institute for Global Health, Beijing Representative Office, China)

Chair, Data Safety Monitoring Board: Jesse Dawson

ClinicalTrials.gov: NCT03790800

Chinese Trial Registry: ChiCTR1900020534

Trial Sponsor: The George Institute for Global Health, Beijing Representative Office

Shanghai East Hospital, Tongji University, China

The First Affiliated Hospital of Chengdu Medical College, China

Date of Document:

Version 1.0 : 17 July 2019

目录

4.1 Schedule of Meetings....5

4.2 Attendance....6

4.3 Declaration of Conflict of Interest....6

4.4 Confidentiality....6

4.5 Copyright....6

4.6 Format of Meetings....6

4.7 Meeting Minutes....7

5.1 Term of Membership....7

5.2 Members....7

I have read and approve this Charter: 9

1. Introduction

INTERACT4 is a multicentre, ambulance-delivered, prospective, randomized, open-label, blinded endpoint (PROBE) study to assess the effects of hyperacute intensive blood pressure (BP) lowering initiated in ambulance setting on (i) functional outcome in patients with acute stroke (ii) safety in patients with confirmed acute stroke and other conditions that were initially suspected as acute stroke (i.e. stroke mimic).

All patients who meet the eligible criteria for the study will be randomised to either:

Intervention group:To commence BP lowering in the ambulance to achieve a target SBP <140mmHg within 30 minutes, and to maintain this level after admission to hospital in those with confirmed acute stroke for the next 7 days (dead or hospital discharge if earlier). A standard treatment regime based on intravenous (IV) bolus of 25mg urapidil administered over 1 minute. For those patients initial SBP >180mmHg, another 25mg urapidil bolus will be given if the BP level persists >150mmHg after 5 minutes. BP monitoring will be done in the ambulance and be recorded every 5 minutes on electronic monitoring charts delivered to hospital staff. Allocation to early intensive BP lowering will be notified to hospital investigators as early as possible by ambulance staff. IV bolus (or maintenance infusion) treatment will continue in the emergency department (ED) for those patients with confirmed acute stoke where it is anticipated that patients will stay until the target SBP is achieved and they are clinically stable. BP lowering treatment would be continued in an acute stroke unit or other monitored facility, in order to maintain a SBP level <140mmHg for the next 7 days. Oral BP lowering agents (depend on local availability) can be used after a stable target BP level reached but it is expected that IV therapy will continue to be required during the initiation of oral antihypertensive therapy, in order to maintain the SBP levels of <140 mmHg. A SBP <130 mmHg is the lower limit for cessation of therapy.

Control Group: To receive BP management according to standard local guidelines (in China, recommended SBP targets are <160mmHg for ICH and <180/100 in patients with AIS who are eligible for reperfusion treatment.

The Primary Outcome is functional shift (improvement) in 7-level mRS scores at 90 days (3 months). in cases of confirmed stroke patients .

Secondary Outcomes include the safety in all randomised patients and:

- *FOR ICH patients, haematoma volume at presentation, 24 hours and relative (>33%) and absolute (6 mL)* growth over 24 hours.
- *For AIS patients*, time to and rate of reperfusion treatment (thrombolysis and/or thrombectomy); and infarct size on MRI within 2 days after stroke onset; frequency of reperfusion treatment (thrombolysis and/or thrombectomy) related sICH according to standard definitions.
- *For all stroke patients*, functional outcomes: death or major disability (mRS 3-6) at 3 months; separately on death and disability at 3 months; death or dependency measured by a shift in NIHSS at 24 hours and 7 days; length of hospital stay; living circumstances; HRQoL according to the EQ-5D at 3 months.

This Charter will define the primary responsibilities of the Steering Committee (SC), its relationship with other trial committees, its membership, and the purpose and timing of its meetings. The Charter will also provide the procedures for selecting SC members and organising SC meetings.

2. Role & Responsibilities of the Steering Committee

The Steering Committee (SC) is responsible for the execution of the study design, protocol, data collection and analysis plan, as well as publications. Furthermore, the SC:

- is the decision-making body responsible for implementing modifications to the protocol, including those that may result from recommendations from the study's Data Safety Monitoring Board (DSMB). Further details are provided in the INTERACT4 DSMB Charter.
- has the right to appoint new members and co-opt others to add to the integrity of the conduct of the study and analyses.
- includes leading scientific experts to be involved in the development, approval, and maintenance of the SC Charter.
- will review study progress and have a lead role in the study analysis and publication of results.
- may also be involved in study site education, for the investigators and staff, on an as needed basis.

3. Steering Committee Organisation

3.1 Selection of Steering Committee Members

The Chair and Deputy Chair of the SC will decide the final membership of the SC as well as any changes to the membership for the duration of the INTERACT4 study. Membership is for the duration of the trial unless the member withdraws their membership or is deemed by the SC Chair and Deputy Chair to be unable to fulfil his/her responsibilities. These responsibilities include, but are not limited to, significant contribution to all aspects of the study design, protocol development, conduct, analysis and publication.

3.2 Qualifications of Steering Committee Members

Members will be healthcare providers or other relevant individuals, statisticians, or epidemiologists with clinical and/or research expertise relevant to the design and conduct of the INTERACT4 study. Documentation of the qualifications will be maintained by The George Institute in the form of curricula vitae for the selected SC members.

3.3 Training of the Steering Committee

Members of the SC will be provided with training on:

- Orientation to the project
- Specific committee objectives

This training will be documented and details kept centrally at The George Institute.

3.4 Steering Committee Chair & Deputy Chair

The specific responsibilities of the SC Chair and Deputy Chair include:

- To preside over SC meetings or delegate to an appropriate designee from the SC
- To facilitate the finalisation and sign-off of the SC Charter and associated documents
- To ensure that an accurate account of SC conference calls and meetings is made and circulated to all SC members along with relevant representatives from the operational team
- To ensure that all decisions made by the committee are communicated to all SC members
- To ensure that high quality is maintained in all aspects of study design, protocol development, conduct, analysis and publication
- To communicate to the DSMB Chair any decisions made or issues found

3.5 Steering Committee Members

The SC members are responsible for the following:

- To participate in all SC teleconferences and meetings
- To participate in discussions related to all aspects of study design, protocol development, conduct, analysis and publication
- SC members will communicate to the SC Chair and Deputy Chair any schedule conflicts, including extended time away from office, which may impact on their ability to be part of the SC
- Provide input to the SC Chair and Deputy Chair regarding specific issues that may need to be addressed by the DSMB
- Ensuring that all materials and information are kept strictly confidential and may not be discussed or disclosed with anyone external to the SC unless specifically authorised in this charter.

Responsibilities of individual members:

- Each individual member is expected to assist in achieving the functions of the project through active contributions. In discussing issues, members will bring their expertise, decisiveness; focus on the future and good humor to bear
- Undertake activity as a representative of their agency/country for the project
- Promote and provide advocacy for the project
- All members of the Committee are required to declare any conflict of interest at all times
- During Steering Committee membership, the member will not reveal any confidential or proprietary information entrusted in the course of their duties
- Upon cessation of membership, and thereafter, the member shall not reveal any confidential or proprietary information, which they obtained while a member of the committee, and may not use or retain, or attempt to use or retain, any such information, documents or data
- During membership, and thereafter, the member will respect the copyright of any information and resources developed under the auspices of the committee as agreed by the full membership

4. Steering Committee Meetings

4.1 Schedule of Meetings

SC meetings will be held following the 6 monthly DSMB meeting and in accordance with the schedule below. The SC Chair and Deputy Chair will have the right to determine the need for additional meetings on an as needed basis:

**Face to face meeting**	Once a year opportunistically held in accordance with the location and timing of the European Stroke Organisation Conference in May or the World Stroke Congress in October of any given year.
**Teleconference**	Once a year either in May or October timed alternately with the face to face meeting
**Other teleconferences or face to face meeting**	Convened as required

4.2 Attendance

- To ensure the ongoing management of core business there is an expectation that Steering Committee members will attend all meetings
- Where this is not possible a proxy from the organisation may be nominated to attend
- A quorum is set at 50% of current membership
- People outside the Steering Committee can be invited to participate in particular discussions on relevant issues by agreement of the Steering Committee.
- Members are required to inform the project manger of their non-attendance at any meeting and notify their nominated proxy prior to the meeting date

4.3 Declaration of Conflict of Interest

All SC members are required to declare any conflict of interest at the beginning of each SC meeting, during the trial and in analysis of results.

4.4 Confidentiality

During SC membership, all members will not reveal any confidential or proprietary information entrusted in the course of their duties. Upon cessation of membership, and thereafter, the member shall not reveal any confidential or proprietary information which they obtained while a member of the committee, and may not use or retain, or attempt to use or retain, any such information, documents or data.

4.5 Copyright

During membership, and thereafter, all information and resources developed under the auspices of the committee shall remain the property of the sponsor.

4.6 Format of Meetings

Prior to each meeting, an agenda and relevant documentation will be circulated to all SC members and other individuals attending the meeting in a non-voting capacity.
The agenda will usually follow the format below:

- Introductions
- Review agenda
- Minutes from last meeting
- Review of actions arising from previous SC meetings
- Overall study status including site status, recruitment, adherence
- Report from the Medical Adjudication Committee (if relevant)
- Report from the DSMB (if relevant)
- Consideration of other items relevant to the study
- Review and summarise new actions from the current meeting
- Any other business
- Plan, date and location for next meeting

4.7 Meeting Minutes

Meeting minutes will be taken by the Project Manager or delegate at each meeting. The minutes will be sent to the Chair and Deputy Chair, for review after a meeting and prior to circulation among the remaining attendants. Following review by the chairs, all SC members with have the opportunity to comment on the minutes within the timeframe of two weeks, after which, the minutes will be finalised. Finalised minutes will be presented for acceptance at the next meeting and filed at The George Institute.

5. Membership

5.1 Term of Membership

- Independent SC members will be invited whose organisation won't take part in this trial
- Members of the Steering Committee will comprise individuals interested in the operations and success of the project
- The committee will have representation from regions participating in the project
- Membership will be for the duration of the trial
- If any member leaves during the course of the trial, the Operations Executive Committee will appoint an appropriate replacement.

5.2 Members

Name	Role	Country	Contact details
Professor Craig Anderson	Chair	Australia	The George Institute for Global Health, Beijing Representitave Office<br>T +86 10 82800577 ext 557<br>M +86 15210347573<br>E :canderson@georgeinstitute.org.cn

Professor Gang Li	Member	China	Shanghai East Hospital, Tongji University, China<br>T: +86-21-38804518 ext 22017<br>M:+86 13621691786<br>E: ligang@tongji.edu.cn
Professor Jie Yang	Member	China	The First Affiliated Hospital of Chengdu Medical College, China<br>M:+86 13678130516<br>E: yangjie1126@163.com
Dr. Lily Song	Member	China	The George Institute for Global Health, Beijing Representitave Office<br>M +86 13916466400<br>E :lsong@georgeinstitute.org.cn
Dr.Hisatomi Arima	Member	Japan	Fukuoka University, Fukuoka, Japan<br>E: harima@fukuoka-u.ac.jp

6. Version Control

Version	Date Finalised	Changes Made
**1.0**	17 July 2019	Original

7. Appendix 1 – Steering Committee Charter Signature Sheet

Members of the Steering Committee undertake their respective roles on the Steering Committee in a volunteer capacity. By signing below, all members agree to comply with the requirements set forth within this Charter and in doing so shall be indemnified by The George Institute.

I have read and approve this Charter:

Name	Signature	Date
Professor Craig Anderson		28-Nov-2019
Professor Gang Li		28-Nov-2019
Professor Jie Yang		28-Nov-2019
Dr. Lily Song		28-Nov-2019
Dr.Hisatomi Arima		28-Nov-2019
