# Supplementary material for: INTEnsive ambulance-delivered blood pressure Reduction in hyper-ACute stroke Trial (INTERACT4): study protocol for a randomized controlled trial
Source: Trials. 2021 Dec 6;22:885. doi: 10.1186/s13063-021-05860-y (PMC8646007; doi:10.1186/s13063-021-05860-y)
Supplement: Supplementary file 4 — Additional file 4.. Informed consent materials [file 13063_2021_5860_MOESM4_ESM.zip › 4.Participant information CF_V3.0_17Jul2019_CN-cleanR1.pdf]

医院名称：____

脑卒中急救车超早期强化降压的研究(INTERACT4)

患者须知及知情同意书

简介

医院正在参加一项临床研究，研究的目的是评估伴随高血压的超早期疑似脑卒中患者在救护车上启动静脉降压的治疗效果。您受邀参加本项研究是因为：(a)您可能患上了急性脑卒中（中风），包括缺血性卒中（脑梗死），是由于血管被血栓阻塞使部分脑组织的血液供应减少；或者是脑出血，是由于血管破裂导致脑内血肿形成；以及(b)您的血压明显升高可能提示不好的预后。

您已经在救护车上进行了随机分组，进入了强化降压组进行强化降血压或者进入了标准降压组遵循目前的诊疗方案。本知情同意介绍简述了本研究的内容，并解释了研究流程和治疗方法。我们希望这些能帮助您决定是否继续参加本研究以便于我们在未来的几个月中继续随访您的康复情况。请仔细阅读本介绍。您可以针对任何感到疑问的地方或需要更多了解的地方进行提问。如果可能，在您做决定是否参加之前，您可以和您的亲属、朋友或者护理者讨论。

参加本项研究是自愿的。您可以自由选择是否参加研究。无论是否参与本研究，都会得到最好的医疗处置。

如果您决定继续参加本项研究，您需要签署知情同意书，代表您：

- 全部理解了所有阅读材料；
- 同意继续参加本研究；
- 同意所述的检查和治疗；
- 同意使用您的个人健康信息。

此介绍和您签署后的知情同意书的复印件将留给您保存。

研究目的是什么？

本研究旨在探索救护车上对疑似脑卒中的患者进行早期降压治疗是否可以增加康复的机会。已有证据表明这样的治疗可以增加急性脑出血患者的康复概率，脑出血是一种脑内血肿形成的严重卒中。理论上越早控制血压，脑内出血就可以越好的控制，就像房间里减少水压以减少管道漏水。早期降压也可能有助于急性脑梗死患者更快进行治疗并减少治疗相关的脑出血风险。但目前对于治疗的潜在获益和风险均缺少足够的证据。

参与这项研究包括哪些活动?

流程

在救护车上通过免除知情或简要知情，您已经被随机分配到强化降压组或常规降压组。强化降压即在 30 分钟内将收缩压降至 130-140mmHg，强化治疗组的治疗方案是静脉注射 25mg 乌拉地尔如果血压在 5 分钟后复测收缩压仍高于 150mmHg，将再注射 25mg 乌拉地尔；后者则是目前救护车上常规血压管理办法，只有当血压非常高（如收缩压≥220mmHg 或舒张压≥110mmHg）时考虑给予降压治疗。

转运至医院后，作为疑似脑卒中患者的常规诊疗包括：

- 收集您的既往病史；
- 临床评估（GCS,NIHSS,mRS)；
- 测量您的血压和心率；
- 收集您的血液检测报告，如生化、血细胞计数及凝血功能报告；
- 神经系统及一般体格检查；

- 心电图检测心律；
- 头颅影像学检查（CT 或 MRI）以明确卒中的类型及严重程度；可能进行 CTA（CT 血管造影）来决定是否需要进行取栓治疗；

这些步骤通常将在入院后完成。不同诊断的患者将得到不同的治疗。如果您患有急性缺血性卒中，您可能符合静脉溶栓药物 rtPA（重组组织型纤溶酶原激活剂或阿替普酶®）和/或需麻醉下的动脉取栓治疗。医生将向您解释这些治疗。脑出血的治疗有所不同。同样的，针对非急性卒中的其他疾病也有相应的治疗。

这些步骤之后，您将决定是否继续参加这个临床研究并随访您今后的健康状况及康复情况。继续参加这项研究有以下操作：

- 您被诊断为急性脑卒中，包括急性缺血性卒中或脑出血；
- 按照在救护车被随机分配的方案，您将在发病 7 天内继续接受积极降压治疗（目标收缩压为 130-140 毫米汞柱）或者目前常规诊疗的血压管理。
- 积极降压治疗包括在最初的几天使用静脉降压药物，随后改为口服降压药物以维持血压在目标水平；
- 如您不是急性脑卒中患者，将按照诊疗常规进行血压管理；
- 您入院时的部分病史信息将被收集用于研究；
- 有关您的治疗方面的部分信息将被用于研究；
- 在您病后的 3 个月（±7 天）研究人员会通过面对面或者电话评估您的健康状况和康复程度，过程中需要您配合 EQ-5D 问卷的完成，以及收集健康相关的花费信息。这个过程约需 20 分钟左右。

- 您的部分医疗信息将与其他病人的信息成组使用于将来的统计分析和科学报告上。这些信息会被一个研究小组进行分析，并不包含任何您的个人身份识别信息。

参与研究有什么风险或获益吗？

可能的获益：

我们希望研究的结果可以改善超急性期脑卒中患者的院前急救措施，促进患者的预后。积极地降低血压具有潜在的益处，比如降低脑出血的血肿体积，以及降低脑梗死血管再通治疗后发生严重脑出血的风险。我们无法保证或承诺您能从参与本研究而得到任何直接的利益。

可能的风险：

乌拉地尔可能引起血管性水肿、荨麻疹、鼻塞、阴茎异常勃起、头晕、恶心、呕吐、出汗、心烦意乱、乏力、心悸、心律失常、心动过速、心动过缓、前胸部紧缩或疼痛等，这些多数由血压迅速下降所致。然而，这种反应通常在几分钟内消失，不需要中断治疗。

偶尔可能出现体位变化导致的血压下降。

过敏反应（如瘙痒、皮肤变红、皮疹等）是罕见的。

个别病例显示口服该药后出现血小板计数下降，但血清免疫学研究尚未证实其因果关系。

其他降压药也可能有副作用。这些副作用一般轻微且少见，减量或者停止治疗后就可能立刻消失。这些副作用包括常见的低血压，头晕、头痛和呕吐（所有这些都是少见的副作用）。同其它所有药物一样，降压药物也可能出现过敏反应，但是这也是很少见的。

接受了积极降压的患者可能出现低血压，从而使一些器官血流降低，导致肾脏或者大脑的损害，但这种风险的几率很低。设计本项研究的医生们曾经研究了几千例自发性脑出血患者使用此降压治疗措施的结果。那一研究显示此降压治疗出现严重副作用的概率非常低。如果您对您使用的药物有任何疑问和不解，请咨询您的医生或者护士。

如果有新的研究内容披露将如何处理?

在研究的过程中，研究者可能会获得降压治疗获益和风险的信息更新。如果出现了这种情况，您将及时从您的医生处获得这些信息并与医生讨论这些信息会有怎样的影响。

必须参加这个研究吗?

您参加任何临床研究都是自愿的。如果不愿意参加本研究，您可以拒绝。如果您决定参加研究但是改变主意了，您可以随时从研究的任何环节中退出。无论您的决定是什么，请您放心这不会影响您的治疗或者与医务人员的关系。当然，如果您从研究中退出后同意继续接受研究随访将对研究结果非常重要。

如果退出研究会怎么样？

如果您决定退出本研究，请尽快联系研究团队中的任何一人并告知他们从上次访视后发生的任何医疗事件及合并用药。这些将有助于研究团队与您进一步讨论健康风险及特殊的要求，而这些可能会影响您的决定。

如果您决定退出研究，研究人员仍然希望能保留已经从您那收集的信息。这将帮助他们确认是否已经正确地计算研究结果。如果您决定退出研究并要求撤回已经收集的信息，请告知研究医生。

保密

研究所收集的有关您的所有信息都将严格保密，且只有经您的许可才会被披露，法律规定的除外。只有 INTERACT4 研究人员、监查员和监管机构以及伦理委员会的代表才能直接查阅参与者的医疗记录。查阅医疗记录是为了核对收集信息的准确性，确保依照本地要求和监管指南开展研究。

研究收集的有关您的医疗记录信息将以电子格式保存在为本研究专门设计的中国境内的数据库内。您的信息将仅由您的姓名首字母缩写、出生日期和研究登记号标识。计算机的所有记录都有密码保护，以电子方式传输的任何信息都将编码，您的身份识别信息将在研究结束后的一段时间内被永久删除，以便保护您的隐私。

研究结果可能在会议或者科学出版物上发表，但是不会由此识别出个体患者。研究中采集的数据中去除身份识别信息的部分未来可能通过一个遵照高标准规则制定的数据共享协议分享给其他的研究者们，由此可以更好的了解脑卒中对患者的影响以及患者对治疗的反应情况，以便于更好的管理这个严重的疾病。

根据当地法律，您有权查阅、要求纠正或删除与您相关的信息。如果您有上述要求，请联系下文列出的研究人员。

对于损害或者并发症的赔偿

如果由于参与本研究导致您受到由研究干预措施导致的损伤或者出现了并发症，您应该尽快和研究医生取得联系，他们将帮助您安排合适的医学治疗。除此之外，当发生由研究干预措施导致的伤害或并发症时，将由研究资助方和相应的保险公司，依据相关保险和赔偿条款，提供相应的免费医疗和补偿。

参加研究需要支付费用吗？

参加研究不需要支付任何费用，同时研究也不会向您支付任何费用。

联系信息

如果您想了解关于这个研究更多的信息或者关于这个研究有任何医学问题，可以随时联系该院的主要研究者：____________________或者下列人员：

姓名：

职位：

电话：

伦理批准和投诉

这项研究已经获得_____________ 伦理委员会的批准。对这项研究有疑问或者相关投诉，可以联系伦理委员会，联系电话：__________________。

这份资料将由您保存

患者须知及知情同意书

……………………………………………………………………………………………………（地址）

我已经阅读并理解了《患者须知及知情同意书》（本材料 1-7 页）关于 INTERACT4 的内容，并被告知将在 3 个月时接受研究人员的随访并且同意其收集信息。

我已经了解这项研究的程序，包括根据目前研究人员掌握的信息得出的所有已知或者预期的不便、风险、不适或者潜在的副作用。我有机会进行提问，并且提出的所有问题均得到满意的回答。我也理解参与这项研究意味着允许研究人员和其他相关人员（患者信息表列出的人员）出于研究目的查阅我的诊疗记录。

我同意被随访，也知道随访的内容是关于我的健康状况、康复程度和健康相关费用信息。

我同意参与本研究，也明白我可以在任何时间退出研究。

- **INTERACT4** □***同意/*** □***不同意***

患者或其代理人姓名（正楷）______________________与患者关系_____________

患者或其代理人签名______________________ 签名日期_________________________

获得知情同意的研究者的姓名（正楷）_________________________________

获得知情同意的研究者签名____________________ 签名日期__________________________

见证者（如有）姓名（正楷）___________________________

见证者（如有）签名___________________________ 签名日期________________________
