# Supplementary material for: INTEnsive ambulance-delivered blood pressure Reduction in hyper-ACute stroke Trial (INTERACT4): study protocol for a randomized controlled trial
Source: Trials. 2021 Dec 6;22:885. doi: 10.1186/s13063-021-05860-y (PMC8646007; doi:10.1186/s13063-021-05860-y)
Supplement: Supplementary file 4 — Additional file 4.. Informed consent materials [file 13063_2021_5860_MOESM4_ESM.zip › 4.Participant information CF_V3.0_17Jul2019_EN-cleanR1.pdf]

Hospital Name：____

INTERACT4 (**INTE**nsive ambulance-delivered blood pressure **R**eduction in hyper-**AC**ute stroke **T**rial)

Participant Information and Consent Form

Introduction

This hospital is currently participating in a clinical trial aiming to determine the effect of very early lowering of elevated blood pressure (BP) in ambulance in patients with suspected acute stroke. You are invited to take part in this study because: (a) you have had a suspected stroke, either ischemic from a clot blocking a blood vessel to reduce blood supply to a part of the brain, or intracerebral hemorrhage from bleeding in the brain; and (b) you also have elevated BP which predicts a poor outcome.

You have been randomised to either intensive BP lowering or to standard care with routine treatment in the ambulance. This Participant Information Sheet outlines the contents of this study and explains procedures and treatments that are involved. We hope this will help you to decide continuing in this study so that we can assess your recovery conditions in the next several months. Please read this information carefully. Ask questions about anything that you don't understand or want to know more about. Before deciding whether to continue, you may to discuss it with your relatives or friends.

Participation in this research is voluntary. It is your choice whether to continue participating in this study. You can receive the best medical care irrespective of whether to continue this study.

If you decide to continue with this study, you will be asked to sign this Patient Consent form. By signing this, you are telling us that you:

- Totally understand what you have read;
- Agree to continue with this study;
- Agree to the tests and treatments that you are prescribed;
- Agree to use your personal and health information as described.

A copy of your signed and dated Participant Information & Consent Form will be given to you to keep.

What is the purpose of this study?

This study is investigating whether the early control of elevated BP in the ambulance for patients with suspected acute stroke will improve the chances of recovery. There is evidence that such treatment can improve recovery for patients with the more serious type of stroke due to bleeding in the brain, acute intracerebral haemorrhage. Theoretically, the earlier the BP be controlled, the better than bleeding in the brain can be reduced, much like reducing pressure in a leaking pipe in a house. Early lowering BP may also assist in the speed of treatment and reduce any bleeding complications associated with the treatment of a stroke due to the other type, acute ischaemic stroke. There is limited information available about the balance of potential benefits and risks of such treatment.

What does participation in this study involve?

Procedures

You have been randomised in this study after a waver or brief consent process in the ambulance, into either intensive BP lowering or standard care with routine treatment. Intensive BP lowing will achieve a systolic BP level to 130-140mmHg within 30 minutes by intravenous bolus of 25mg Urapidil and another 25mg Urapidil bolus will be given if the systolic BP level persists above 150mmHg after 5 minutes. Standard care is the usual BP management method of which will be considered to be given BP lowing only for those with persistent very high systolic BP (e.g. ≥ 220mmHg) or diastolic BP (e.g. ≥ 110mmHg) in the ambulance.

After admitted to hospital, as part of your usual care of suspected stroke, the following procedures are likely to been conducted:

- Collect information about your medical history;
- Clinical assessment（GCS，NIHSS，mRS）；
- Measure your BP and heart rate;
- Collect the reports of blood test, such as biochemistry, cell count and clotting;
- A neurological and general physical examination;
- Examine your heart rhythm using an ECG;
- A scan of your brain (either a computerised tomography [CT] or magnetic resonance imaging [MRI]) to confirm the type and severity of the stroke; and you may have additionally a CT angiography to decide whether you require any treatment to remove a clot in the brain;

Generally, these procedures will be completed after your arrival at hospital. Your treatment will be based on your specific diagnosis. If you have an acute ischemic stroke confirmed, you may be eligible to receive clot-busting medication called rtPA (recombinant tissue plasminogen activator or Actilyse®) and/or removal of a clot via an artery to the brain for which you will require anaesthetic. Your doctor will explain this to you. Treatment of any haemorrhage is different. Similarly, there are different treatments for particularly diagnoses that are not due to acute stroke.

After these procedures, you will be asked whether continue staying in this study and following-up of your health condition and recovery information. You will continue in this study for the following procedures:

- If you are diagnosed with acute stroke, whether ischemic or intracerebral haemorrhage;
- you will continue to receive BP lowering treatment according to your randomized allocation, either for a lower level of systolic in the range of 130-140 mmHg for the next 7 days or BP management according to routine clinical practice at present.
- Intensive BP lowering treatment involves the use of intravenous medication for the first few days and followed by oral (taken by mouth) tablets to maintain the BP at the target levels;
- If you were diagnosed with non-stroke, you will be received other BP management and treatment according to clinical routine practice;
- Some information about your medical history will be collected for this study;
- Some information about your treatment will be used for this study;
- In about 3 months (±7 days), you will be contacted face to face or via telephone for a short interview (about 20 minutes) by an investigational staff to evaluate the condition of your health and the level of recovery(you are required to complete a questionnaire called EQ-5D) and health related cost during this interview;

Some of your medical information except your personal information will be used with the other participants, to be examined as a group for future statistical analysis and scientific reports, undertaken by an experienced research group .

What are the potential benefits and risks?

Potential benefits

We hope the results of this study will improve the pre-hospital treatment and outcomes for people who experience with acute stroke in the future. The potential benefits of very early

intensive BP lowering are reduce bleeding in the brain from intracerebral haemorrhage or thrombolysis treatment of acute ischemic stroke. However, we cannot guarantee that you will receive any direct benefits from participating in this study.

Potential risks

Urapidil may cause angioedema, urticarial, nasal obstruction, mentulagra, headache, dizziness, nausea, vomiting, sweating, dysphoria, weakness, palpitation, arrhythmia, tachycardia, bradycardia, upper chest constriction or pain, etc., which may be caused by rapid reduction of blood pressure. However, such reactions generally disappear within a few minutes, and it is unnecessary to interrupt the treatment.

Blood pressure decrease caused by change of position shows occasionally.

Allergic reactions (such as pruritus, reddening of skin, rashes, etc.) are rare.

Very individual cases show blood platelet count decrease when orally taking this drug, but serum immunological studies have not verified their causal relationship.

Other BP lowering drugs may also have side-effects which are generally mild and infrequent, and can be resolved immediately by reducing or stopping the treatment. These side-effects include hypotension (low BP) which is moderately common, dizziness, headache and vomiting (all of which are rare). As with any medication, an allergic reaction to BP lowering drugs is possible, but this is rare as well.

Other potential risk of BP lowering is that it could decrease blood flow to an organ of the body such as kidney or brain, which can cause some damage. However, the risk of this occurring is very low. The doctors who designed this study have investigated thousands of spntaneous intracerebral hemorrohage patients who have received BP lowering treatment that is being used in this study and the risk of serious side-effects of the treatment is very low. If you have any questions or concerns about your treatment, please communicate with your doctor or nurse.

What if new information arises during this study?

During this study, new information about the risks and benefits of treatment may known to the researchers. If this occurs, you will be told about this new information in a timely manner and your doctor will discuss whether this new information affects you.

Do I have to stay in this research study?

Participation in any study is voluntary. If you do not want to take part in, don't have to. If you decide to take part in but change your mind later, you are free to withdraw from this study at any time. Your decision on whether to take part in or to take part in and then withdraw will not affect your routine treatment and your relationship with those treating you or hospital in which you receive your care. However, it is very important if you can try to stay in this study, as your withdrawal will affect the quality of this study a lot.

What if I withdraw from this research study?

If you decide to withdraw from the study, please notify a member of the research team as soon as possible and inform them of any medical problems you experienced or medications you have taken since the last study contact. This will allow the research team to discuss further health risks or special requirements that may have influenced your decision.

If you decide to leave the study, the researchers would like to keep the health information about you from which has been collected. This will help them to make sure whether the results of the research can be measured properly. If you decide to withdraw and do not wish for your health information that has already been collected to be used, please notify the hospital study staff.

Confidentiality

All the information collected from you for this study will be treated confidentially,except as otherwise stipulated by law. And only the INTERACT4 researchers, monitors , the representatives of regulatory authorities and the ethics committee can have direct access to it. Access is required to check the accuracy of the information collected and to ensure that this study is being carried out according to local requirements and/or regulatory guidelines.

All information collected from you for the study will be stored electronically in a database in China specially designed for this study.Your information will be identified by your initials, date of birth and a study registration number only. Any information transferred electronically will be coded to protect your confidentiality. All computer records will be password protected. Your identifiable information will be deleted permanently for a certain period of time after the end of the study to protect your personal privacy.

The study results may be presented at a conference or in a scientific publication. You will not be personally identified in any reports or publications resulting from this study. Some de-identifiable data may be shared with other researchers as part of data-sharing research that

follows high-standard policies to better understand how different people are affected by stroke and respond differently to treatment, in order to better direct the management of this serious condition.

You have right of access, correction and delete information about you in accordance with the local law. Please contact one of the researchers named at the end of this document if you would like to have below request.

Compensation for injuries or complications

If you suffer any injuries or complications due to the interventions of this study, you should contact the study doctor as soon as possible, who will assist you to have appropriate medical treatment. In addition, when you suffer any injuries or complications caused by this study interventions,you may be able to receive medical treatment required to treat the injury or complication, free of charge, according to relevant insurance and compensation clauses in the insurance coverage purchased by the researchers for this study.

Will taking part in this study cost me anything?

Participation in this study will not cost you anything and your will not receive any payment.

Contact Information

If you want any further information about this study or if you have any medical problems which may be related to your involvement of this study, you can contact with the principal investigator on [____________________________] or any of the following person:

Name: ____
[INSERT NAME OF STUDY COORDINATOR OR KEY RESPONSIBLE PERSON]

Role: ____
[INSERT ROLE OF KEY RESPONSIBLE PERSON]

Telephone: ____
[INSERT TELEPHONE NUMBER OF KEY RESPONSIBLE PERSON]

Ethics Approval and Complaints

This study has been approved by the Ethics Committee of ____. Any person with concerns or complaints about the conduct of this study should contact the Ethics Committee on ____
[Ethics committee contact details]

This information will be kept by you

Participant Information and Consent Form

………………………………………………………………………….. [ADDRESS]…

I have read and understood the Participants Information Sheet (pages 1-6 of this material) about the INTERACT4 study and was told that I would be followed up by the researchers at 3 months and agreed to collect the information

I have understood the procedures of this study, including all known or expected inconveniences, risks, discomforts, or potential side-effects based on information available to researchers. And I had opportunity to ask questions, and all the questions raised were answered satisfactorily. I also understand that my involvement in this study will allow researchers and other stakeholders (listed on patient information sheet) to access my medical records for research purposes.

I agree to a later interview to provide information about my health, recovery and health related service costs.

I am free to choose whether to take part in the research and understand that I can withdraw from this research at any time.

INTERACT4 ☐ **Agree / ☐ Disagree**

Participant's or legal representative's Name ____________________________________
Relationship with Participant ____

Participant's or legal representative's Signature __________________________

Date____________________

Informed Consent Researcher's Name ____

Informed Consent Researcher's Signature_________________ Date__________

Witness Name (if applicable)________________________________

Witness's Signature(if applicable)______________________Date_____________________
