# Supplementary material for: INTEnsive ambulance-delivered blood pressure Reduction in hyper-ACute stroke Trial (INTERACT4): study protocol for a randomized controlled trial
Source: Trials. 2021 Dec 6;22:885. doi: 10.1186/s13063-021-05860-y (PMC8646007; doi:10.1186/s13063-021-05860-y)
Supplement: Supplementary file 5 — Additional file 5.. [file 13063_2021_5860_MOESM5_ESM.pdf]

院前急救知情同意书

Informed consent for pre-hospital first aid

一、基本情况

Basic Situation

1、患者姓名__________________ 男/女 年龄_________岁
Patient's Name *Male/Female* *Age*

2、患者亲属姓名 ______________________ 与患者关系 ______________________________
Name of Relatives *Relationship with Patient*

3、运送性质： 送院/转院/回家 送往__________________________________
Purpose: *Send to hospital/Transfer to another hospital/Home*

二、风险告知

Risk notification

1、患者病情危重，在搬运或运送途中随时可能发生病情加重而危及生命，患者（亲属）表示理解，要求搬运及运送。
The patient is in critical condition and may be aggravated and life-threatening at any time during transportation. The patient (relative) understands the risk and requests transportation.

2、遵循患者（亲属）医院送往____________________医院，患方自愿承担转送风险。
Follow the patient's (relative) will to transfer the patient to_______________ Hospital and patient (relative) is willing to take the consequences.

3、患者病情危重，需遵循就近能力原则送往医院，但患者（亲属）坚持要求送往自己指定的医院____________________，由此发生病情加重及危及生命等转运风险，患方同意自负。
The patient is in critical condition and needed to be transferred to the nearest hospital. But patient (relative) insists on being transferred to Hospital and is willing to take the consequent risk of aggravation and life-threatened condition relatively.

4、由于患者（亲属）拒绝急救医生在现场或运送途中做相关的___<u>检查/救治</u>_____，而引起病情加重及危及患者生命的后果，患者（亲属）愿风险自负。
Patient(relative) refuses the <u>test/treatment</u> conducted by the ambulance doctors on scene or during transferring and is willing to take the consequent risk of aggravation and life-threatened condition.

5、患者（亲属）在救护车到达现场后，不愿意送医院做进一步诊治，虽经急救医生劝说并告知可能产生的风险，但患者（亲属）仍予拒绝，并自愿承担风险。
When arriving, patient(relative) refuses transferring to hospital after knowing the risk and willing to take the consequences.

6、救护车到达现场时患者呼吸、心跳已停止，已向亲属告知患者目前情况。
When ambulance arrives, the patient has lost the vital sign and the patient's condition was informed.

7、救护车到达现场时患者呼吸、心跳已停止，亲属拒绝急救医生进行抢救，若有风险同意自负。
When ambulance arrives, the patient has already lost the vital sign. Relative refuses the treatment and is willing to take consequences.

8、患者（亲属）拒绝急救人员抬抱，要求自行走上急救车，经急救人员劝说并告知了可能产生的风险，患者（亲属）表示理解并自愿承担风险。
Patient (relative) refuses to be lifted and insists to walk onto the ambulance after knowing the risk and willing to take the consequences.

9、告知患者（亲属）目前疑似脑卒中，收缩压≥150mmHg，可能需要尽早降压治疗。患者（亲属）是 / 否 同意参与一项关于早期进行静脉降压治疗的临床研究并记录其相关数据。
This patient is suffering from a suspected acute stroke with currently SBP≥150mmHg, and immediate and quick antihypertensive treatment may be necessary. I agree to take part in a clinical trial related to early BP reduction with intravenous blood pressure lowering agency and get my medical information recorded. (YES / NO)

10、其他表述____
Other conditions

上述“基本情况”属实，有关_________圈出的“风险告知”项目已告知，患者（亲属）已充分了解并签字确认。
The above "basic situation" is true, and patient (relative) signed it with fully understand the circled _______ risk notification.

患者（亲属）签名___________________ __________________________________
Patient(relative) *Doctor*

_________年____月____日___时___分 _________年____月____日___时___分
_ _ : _ _(hh:mm)_ _ / _ / _ _ _ _(dd/mm/yy) *_ _ : _ _(hh:mm)_ _ / _ / _ _ _ _(dd/mm/yy)*
